# Connecting Material Characteristics with System Properties for Membrane-Based Direct Air Capture (m-DAC) Using Process Operability and Inverse Design Approaches

**DOI:** 10.1021/acs.iecr.4c04553

**Published:** 2025-04-10

**Authors:** Vitor Gama, Deepanjali Roy, Fernando V. Lima, Oishi Sanyal

**Affiliations:** Department of Chemical and Biomedical Engineering, West Virginia University, Morgantown, West Virginia 26506, United States

## Abstract

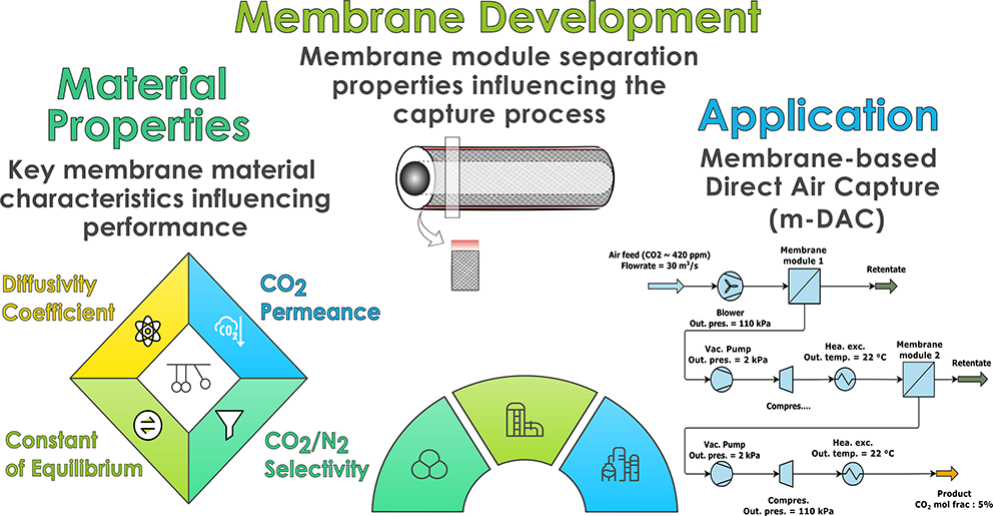

This paper presents a process modeling approach for a two-staged membrane-based direct air capture (m-DAC) process, considering material characteristics, membrane separation, and system properties. m-DAC is a negative emissions technology for capturing dilute CO_2_ from air. Its continuous and modular nature could reduce economic challenges compared to sorption-based processes, which require costly regeneration. Facilitated transport membranes, with specialized CO_2_ carriers, offer higher performance than traditional sorption-diffusion membranes. Their key properties-the CO_2_ apparent diffusion coefficient () and equilibrium constant (*K*_eq_)-determine membrane separation properties such as CO_2_ permeance and CO_2_/N_2_ selectivity. This work maps these inputs to feasible output spaces such as for CO_2_ recovery, purity, and capture cost. Additionally, inverse design is used to determine the required membrane properties for target system outcomes. Overall, this study provides a framework for membrane researchers to design cost-effective, scalable m-DAC solutions.

## 1 Introduction

Climate change is one of the most critical global challenges of the 21st century, primarily driven by anthropogenic carbon dioxide (CO_2_) emissions. Among the various strategies to mitigate CO_2_ emissions, direct air capture (DAC) technologies have gained significant attention for their potential to remove CO_2_ directly from ambient air.^1−3^ However, capturing CO_2_ from the atmosphere is inherently challenging because of its very low concentration (approximately 420 ppm), requiring the development of highly efficient materials and processes capable of selectively extracting CO_2_ from such a dilute source.^4^

Membrane-based DAC (m-DAC) has emerged as a promising approach owing to its modularity, energy efficiency, and scalability potential. Traditional membrane technologies have been successfully applied in various gas separation processes, including CO_2_ capture from flue gas and natural gas streams. However, expanding their application to DAC introduces unique challenges, primarily due to the extremely low concentration of CO_2_ in the atmosphere, which demands membranes with exceptional selectivity and permeance.^5^

To address these challenges, understanding for optimizing the transport mechanisms within membranes is crucial. Two primary gas transport mechanisms are considered in membrane technologies: the solution-diffusion (SD) mechanism and the facilitated transport (FT) mechanism. The SD mechanism, commonly employed in dense polymer membranes, relies on the dissolution and diffusion of gases through the membrane matrix, driven by concentration gradients.^6^ While effective for certain applications, the low solubility of CO_2_ at ambient concentrations limits the efficiency of SD membranes for DAC, necessitating membranes with unrealistically high selectivity and permeance.

In contrast, the facilitated transport mechanism offers a more promising route for DAC. FT membranes (FTM) incorporate carrier agents, such as amines or metal ions, that reversibly bind with CO_2_, significantly enhancing their solubility and selective transport through the membrane. This mechanism enables high CO_2_/N_2_ selectivity even at low partial pressures, making FT membranes suitable for capturing atmospheric CO_2_.^7^ Amine-based FTMs have gained significant attention for CO_2_ separation due to their ability to enhance transport through reversible reactions between CO_2_ and amine carriers. FTMs leverage carriers like primary, secondary, or sterically hindered amines to facilitate CO_2_ movement via the ″hopping″ mechanism. This approach allows for high CO_2_ permeability and selectivity without being constrained by the permeability-selectivity trade-off observed in traditional solution-diffusion membranes.^8^ Previous studies have explored CO_2_/N_2_ separation using FTMs with fixed-site amine carriers, including high-molecular-weight polyvinylamine (PVAm) membranes and polyethylenimine/poly(vinyl alcohol) (PEI/PVA) blend membrane and thin-film composite membranes with tertiary amine functionalities.^9−11^ Other works have also explored CO_2_/N_2_ separation by amine mobile site carrier-containing membrane.^8,12−14^

Recent advances in computational techniques, particularly machine learning and process system analyses, have opened new avenues for accelerating material development in membrane technologies. Machine learning algorithms can analyze large data sets from simulations and experiments to identify patterns and predict material properties that lead to optimal performance. By integrating these computational tools with process operability frameworks, we can gain a comprehensive understanding of how material properties influence the overall feasibility and robustness of m-DAC processes under varying operational conditions.^15^

**Figure 1:**
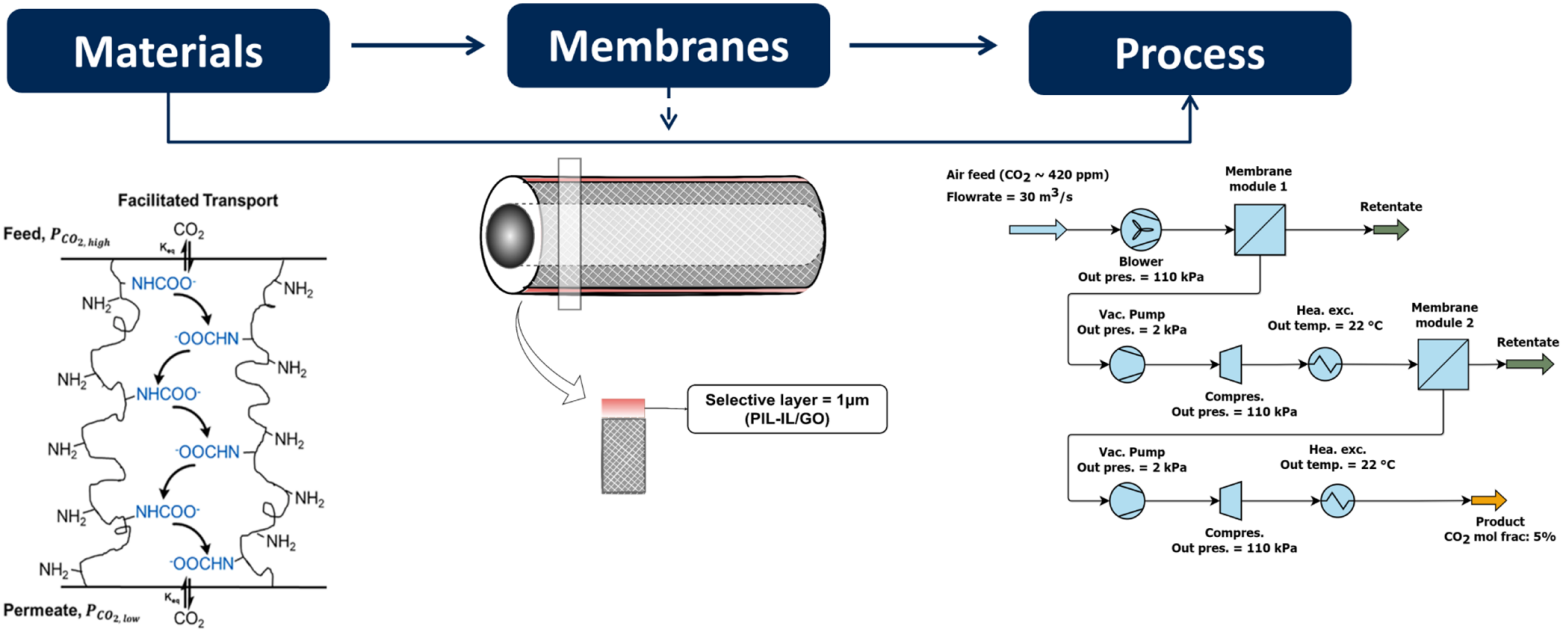
Summary of the work connecting material properties, membrane design and process design. Reproduced or adapted with permission from Xu et al.^16^ Copyright [2025] [Elsevier].

In this work, a novel computational approach is explored for development and optimization of materials for FTMs for m-DAC applications. A combination of computational simulations and machine learning methods is employed to search the space of key membrane properties such as carrier diffusivity and reaction equilibrium constants. The integration of these findings with process operability studies aims to identify optimal material property ranges that ensure high performance and robustness in the m-DAC process. Figure 1 shows a schematic summary of this work that connects membrane material properties to the final system properties. This work builds on our prior research^5^ which specifically describes the design of a m-DAC process to concentrate the ambient CO_2_ (420 ppm) 125 times to produce a low-purity (5%) CO_2_ product. This is in contrast with the typical high-purity CO_2_ concentrations that other DAC technologies (sorbent, solvent based) produce. As prior research^5,17^ has shown, producing high-purity (>90%) CO_2_ with m-DAC would require a multistage operation, making the technology cost-prohibitive. With this rationale, the CO_2_ purity of the permeate stream was set to 5%, and as discussed in our prior paper, such low-purity CO_2_ streams can be used for applications such as indoor farming and algae production.^18^

This approach represents a significant advancement in the field of membrane-based CO_2_ capture from air, providing a systematic framework for the accelerated discovery of new materials tailored for DAC applications. The integration of computational material discovery with process operability analysis accelerates the identification of promising membrane materials while ensuring their practical viability in real-world applications.

## 2 Background

### 2.1 Transport through Membrane Materials

Permeability is directly related to the gas permeation rate through the membrane, or flux (*J*), as well as the thickness of the membrane (*l*), and the partial pressure difference across the membrane (Δ*p*), which serves as the driving force for separation.
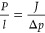
1

In cases where the membrane thickness is not accurately known, permeability is presented as gas permeance (*P*^′^), measured in gas permeation unit (GPU) (10^–6^ cm^3^ STP·cm^–2^·s^–1^·cmHg^–1^):
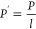
2

The flux, *J*_*i*_, of a gas component *i* through a membrane is given by:
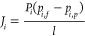
3where *P*_*i*_ is the permeability of gas *i*, *p*_*i*,*f*_ and *p*_*i*,*p*_ are the partial pressures of gas *i* on the feed and permeate sides of the membrane, respectively, and *l* is the thickness of the membrane selective layer.^19^

#### 2.1.1 Solution-Diffusion Mechanism

The solution-diffusion model describes the transport mechanism for nonporous (dense) membranes. This process can generally be categorized into three steps:

1. Gas molecules are adsorbed onto the membrane surface on the upstream side.
2. The gas molecules diffuse through the polymer matrix.
3. The gas molecules desorb from the downstream side.

Based on the SD model, separation is not solely determined by diffusion; it also depends on the physicochemical interactions between the various gases and the polymer. Gas separation is, therefore, described by both the solubility of specific gases within the membrane and their diffusion through the dense polymer matrix. The relationship between permeability, solubility, and diffusivity can be expressed as follows:
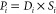
4where *P*_*i*_ is the permeability coefficient of *i*, measured in Barrer (10^–10^ cm^3^ STP·cm·cm^–2^·s^–1^·cmHg^–1^). *D*_*i*_ represents the diffusion coefficient (cm^2^·s^–1^), which quantifies the mobility of *i*, while *S*_*i*_ denotes the solubility coefficient (cm^3^ STP·cm^–3^·cmHg^–1^).

The solution-diffusion flux (*J*_SD_) is therefore
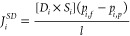
5

The separation performance of solution-diffusion membranes is often constrained by the trade-off between permeability and selectivity. As permeability increases, selectivity tends to decrease, limiting the efficiency of CO_2_ separation.^20^ This relationship is represented by Robeson’s upper bound, which defines the limits of performance for polymer-based membranes.^21^

#### 2.1.2 Facilitated Transport Mechanism

Facilitated transport in membranes is a mechanism used to enhance the selective permeation of a target gas, such as carbon dioxide, through a membrane. Unlike the traditional solution-diffusion mechanism, where gases dissolve into the membrane and diffuse across a concentration gradient, facilitated transport introduces an additional mechanism through the use of carrier agents embedded within the membrane.

The carrier agents are specific moieties that can reversibly bind with the target gas. These carriers are often amines, metal ions, or other reactive agents that selectively bind to CO_2_ molecules. The reaction between CO_2_ and the carrier agents forms a temporary complex. This reaction is reversible, where CO_2_ is released on the permeate side of the membrane. This reversible binding significantly increases the solubility of CO_2_ in the membrane, leading to much higher permeation rates compared to gases that do not interact with the carrier, such as nitrogen (N_2_). The carrier agents are only selective toward CO_2_, with little or no affinity for the cocomponents of the mixture such as N_2_, O_2_, etc. N_2_ and O_2_ therefore permeate through the membrane via the usual sorption-diffusion mechanism, enabling the membranes to achieve high CO_2_/N_2_ selectivities.^8,22^

At lower partial pressures, the facilitated transport mechanism is dominant due to the interaction of gas molecules with carriers within the membrane. However, once the carrier become saturated, the mechanism transitions to solution-diffusion mechanism. So, the overall flux in FTMs, *J*_total_, is the sum of the fluxes from these two mechanisms: the facilitated transport flux (FT) and the solution-diffusion flux (SD). The facilitated transport term dominates at low partial pressures, whereas the solution-diffusion term becomes more significant at higher partial pressures.

The total flux can be expressed as
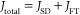
6where *J*_SD_ is the solution-diffusion flux, modeled by eq 5 and *J*_FT_ is the facilitated transport flux.

The facilitated transport flux is mathematically represented by the following:
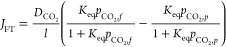
7in which,  is the lumped coefficient related to the apparent diffusivity of the amine carrier (e.g., carbamate) and the concentration of amines sites in the membrane, as described by Xu et al. (2023). *K*_eq_ is the equilibrium constant for the reaction between CO_2_ and the amine carrier.

A number of FTM models have been described in prior literature, a detailed review of which can be found elsewhere.^7^ However, the model developed by Xu et al. (2023) is one of the simpler models which also describes the physical mechanism reasonably well. Unlike complex models, it allows the regression of kinetic and thermodynamic parameters directly from experimental data. This particular model approach makes it useful for capturing the fundamental dynamics of CO_2_ facilitated transport and provides insights into practical membrane performance under varying conditions.

### 2.2 Process Operability Framework

The concept of process operability integrates design and control objectives in the early stages of process development to ensure operational feasibility and efficiency. Operability analysis is based on defining critical sets of input and output variables. These sets are as follows:

- **Available Input Set (AIS)**: The feasible ranges for manipulated or design variables, denoted as .
- **Achievable Output Set (AOS)**: The attainable ranges of output variables, denoted as , given the AIS and the process models.
- **Desired Input Set (DIS)**: The input ranges needed to achieve target output ranges.
- **Desired Output Set (DOS)**: The operational targets specified for output variables.

These sets are determined using the governing equations of the process model *M*:
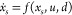
8
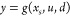
9
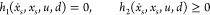
10where  represents state variables,  are inputs,  are disturbances, and *h*_1_, *h*_2_ represent equality and inequality constraints, respectively.^23,24^

Operability can quantify the ability of the process to meet output requirements under input and disturbance constraints, often represented by the *Operability Index (OI)*.^25^ This index measures the overlap between the DOS and the AOS, providing insight into design modifications to enhance operability.

#### 2.2.1 Inverse Design in Operability

Inverse design employs the principles of operability in a reverse framework to determine the necessary input configurations (DIS) to achieve specified output requirements (DOS). This approach is particularly valuable in scenarios where the relationships between input and output variables are nonlinear and computationally intensive to evaluate. Inverse design integrates optimization techniques and mathematical tools such as the implicit function theorem to map the DOS back to a feasible Desired Input Set (DIS*).^26,27^

The DOS represents the range of outputs that satisfy process performance criteria, often constrained by operational goals, environmental standards, and product quality requirements. In the context of membrane-based systems, examples of DOS include target CO_2_ purity and recovery levels.^5^ The feasibility of the DOS is determined by the intrinsic limitations of the process, as captured by the AOS. Similarly, the DIS encompasses the input configurations necessary to achieve the entire DOS under realistic disturbance scenarios.

A feasible DIS, DIS*, is defined as the subset of input parameters that satisfy all physical, operational, and economic constraints of the system. These constraints ensure that the process operates safely, sustainably, and in an economically viable manner.^28^

The intersection between the DIS and the AIS defines the operational space where the process can meet its performance targets. Mathematically, the relationship can be expressed as
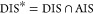
11where the DIS* must also ensure compatibility with disturbances and constraints such as pressure, temperature, and material limitations. The inverse design problem is typically formulated as an optimization task:
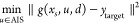
12subject to the constraints in eq 10. Here, *y*_target_ ∈ DOS represents the desired output vector, while *u* is the input vector within the AIS. This optimization seeks to identify input points *u* ∈ DIS*, that achieve or closely approximate the desired output within the allowable tolerances.^28^

Automatic differentiation (AD) tools, such as JAX, have been employed to efficiently compute the Jacobian and Hessian matrices of the process model *M*.^27^ These derivatives are critical for evaluating the sensitivity of outputs to inputs and ensuring the robustness of the optimization process. The implicit function theorem is used to establish the inverse relationships between inputs and outputs, facilitating the path integration necessary for solving eq 12.

Besides AD, Machine learning (ML) can enhance operability analysis by approximating complex, high-dimensional models with surrogate models. Gaussian Process Regression (GPR) and Kriging have been employed to replace first-principles models, enabling faster calculations while maintaining accuracy. These surrogate models support both forward and inverse mappings, expanding the applicability of operability methods to nonlinear and modular systems. The operability framework ensures that the system operates within feasible input ranges while achieving desired performance. ML-based surrogates reduce computational effort, facilitating the exploration of design spaces for membrane optimization.^15^

## 3 Methods

### 3.1 Two-Stage m-DAC Simulation Setup

**Figure 2:**
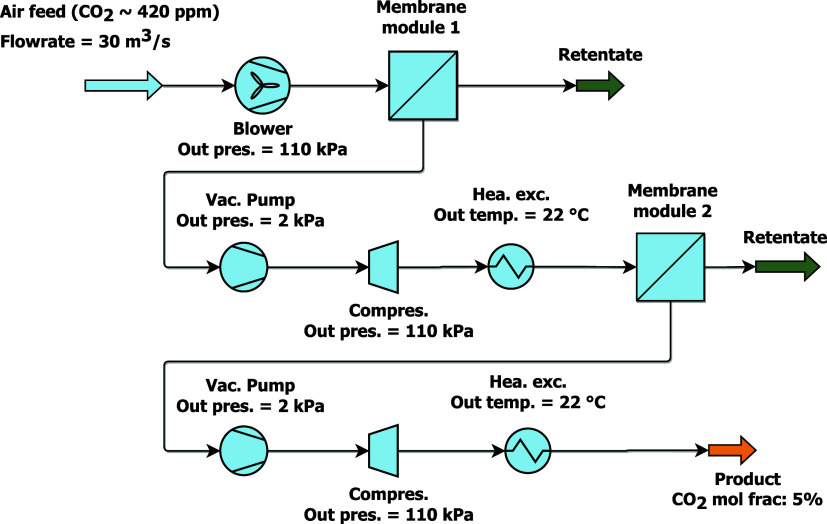
m-DAC process flow diagram.

Building on our previous work on low-purity CO_2_ production via m-DAC,^5^ the capture process was simulated using AVEVA Process Simulation (APS) as a 2-stage separation, as illustrated in Figure 2. The membrane first-principles model is derived from the work of Bishop and Lima (2020) and is described in Section 1.3 of the Supporting Information.^29^ Each module operates with the permeate being vacuumed (2 kPa), while the feed is at atmospheric conditions (110 kPa).^17^ While such low vacuum levels could be operationally challenging, they are required to ensure a sufficiently high pressure ratio across the membrane. Studies, including process simulations, have explored the feasibility of using membranes for DAC and found that employing low permeate-side pressures is a key requirement for achieving sufficient CO_2_ enrichment and maintaining a viable separation process. For instance, Fujikawa et al. (2021)^17^ conducted a process simulation analysis that identified optimized pressure conditions for multistage membrane separation and found that low permeate-side pressures (2–5 kPa) were required to achieve a 1000-fold preconcentration of CO_2_ from atmospheric levels. These low-pressure conditions significantly improve the driving force for CO_2_ transport across the membrane and help maintain reasonable membrane module sizes while ensuring the capture process remains energy-efficient.

For the sake of simplicity, the transport assumptions for this multistage separation follow eq 3. The membrane intrinsic properties values, shown in Table 2, were considered to be the same as the values reported by Lee et al. (2022), seeking to analyze how enhanced properties improve the capture, even though the flux across the membrane is considered a less rigorous mechanism. The simulation operating conditions such as feed flow rates, outlined in Table 1, were kept constant as the intrinsic properties values were manipulated to collect the permeate purity of CO_2_ and calculate its recovery. The effects of processing parameters such as feed flow rate (0.3–300 m^3^/s) and discussions on stage cuts and pressure ratio effects have been reported in the authors’ prior work.^5^

**Table 1:** Simulation Operating Conditions

Parameters	Value
Feed flow rate [m^3^/s]	30
Feed pressure [kPa]	110
Permeate pressure [kPa]	2
Membrane configuration	Hollow-fiber
Flow mode	Countercorrent
Feed composition [mol %]	
Nitrogen	0.79
Oxygen	0.21
Carbon dioxide	0.000420

The presence of humidity was not considered in either the previous study or in this work. The process simulation operating conditions and membrane characteristics are summarized in Tables 1 and 2.

**Table 2:** Membrane Modules Design Parameters

Parameters	Membrane module 1	Membrane module 2
Number of fibers	1 × 10^6^	280,000
Fiber ID [μm]	200	200
Fiber OD [μm]	300	300
Selective layer thickness [μm]	1	1
Fiber length [m]	5	5
Shell OD [m]	5	5
Permeance [GPU]		
CO_2_	2100	128
N_2_	2	2
O_2_	8	8
Selectivity		
CO_2_/N_2_	1100	68
CO_2_/O_2_	265	17

Although the values of the shell diameter (5 m) and length of the module (5 m) are higher than usual, in principle, the overall area could be split into 2 or more parallel membrane modules to accommodate the total feed. Such arrangements are not considered in this paper to limit its scope but this will be considered in our future work. The assumption regarding the 1 μm selective layer is based on the experimentally demonstrated membrane that this study is based on ref (30) reported by Lee et al. (2022). It should be noted that the original paper described a flat sheet membrane, however, in the present work we consider a hollow fiber configuration where only the selective layer (1 μm) is made of the IL.

In this study, three case studies were designed to capture the influence of the membrane intrinsic properties on CO_2_ capture. The data collection was set up to cover a wide range of possible membrane performances using the operability approach. The simulation operating conditions, outlined in Table 1, were kept constant as the intrinsic properties values were manipulated utilizing the *Opyrability*(31) library in order to collect the CO_2_ permeate purity and calculate its recovery. Since this is a two-step separation process, the search space for the properties of each module was approached differently. In the first module, the air entering is at DAC conditions, while the inlet of the second module has higher CO_2_ concentrations, which deteriorates the performance of FTMs due to carrier saturation. Once saturated, the facilitated transport mechanism contribution to the overall flux, *J*_total_, becomes negligible and the solution-diffusion mechanism dominates the transport.^7^Table 3 outlines the designed AIS for each case study.

**Table 3:** Mapping of AIS and DOS for Different Cases

Cases	AIS/Inputs	Desired Output Set (DOS)
**(1)** – 1st Module Impact on Recovery and Purity	CO_2_ permeance: 100–2 × 10^4^	CO_2_ purity: 1.5–2.1%
	CO_2_/N_2_ selectivity: 8–2000	CO_2_ recovery: 10–40%
**(2)** – 1st Module Impact on Capture Cost and Purity	CO_2_ permeance: 100–2 × 10^4^	CO_2_ purity: 2–20%
	CO_2_/N_2_ selectivity: 8–2000	Capture operating cost: 3000–5000
		$/tonCO_2_
**(3)** – 2nd Module Impact on Recovery and Purity	CO_2_ permeance: 100–2 × 10^3^	CO_2_ purity: 4–14%
	CO_2_/N_2_ selectivity: 8–200	CO_2_ recovery: 99–100%

The DOS bounds for Case 1 seek to guarantee the operation of the first stage as close as possible of its separation limit (≈2%), while trying to maintain the recovery at the maximum possible at this purity level. As discussed in Gama et al. (2024),^5^ a trade-off relationship exists between recovery and purity. For Case 2, the proposed DOS focuses on finding the most suitable properties for a cost-effective capture. In this case, the lower capture operating cost is sought to be in between $3000/tonCO_2_ and $5000/tonCO_2_. Regarding CO_2_ purity, a wide range of purities is considered for the DOS. Finally, in Case 3, the specific recovery of the second module shows values close to the upper bound (100%). For that reason, the DOS focuses in the highest values achievable. In terms of CO_2_ purity, the range proposed seek to extend from the minimum purity desired (5%) up until the maximum purity reached by the second stage (≈14%). It should be noted that the purpose of this paper is to primarily demonstrate the process operability methodology involved in identifying the desired input parameters leading to these DOSs. The DOS can be easily changed to cover a different region of operation, and the methodology discussed here would still be useful to identify the corresponding input parameters.

### 3.2 Machine Learning Assisted Inverse Design

To avoid the increased computational time and effort associated with full simulations and to leverage the operability approach, similar to Alves et al. (2022),^15^ surrogate models were trained using simulation data to capture each study’s behavior. This method bypassed the simulation platform solver, which could hinder the performance of the nonlinear programming (NLP)-based approach, while still capturing the first-principles phenomena of the 2-staged separation. The data was split in a 25/75% distribution for training and validation and the regression metrics for each parameter was added to the caption of the respective parity plot, as well as to Figures S6–S8.

#### 3.2.1 Case Studies—CO_2_ Permeance and CO_2_/N_2_ Selectivity Impacts on Capture

##### 3.2.1.1 Case 1: Recovery and Purity Outputs of the First Membrane Module

**Figure 3:**
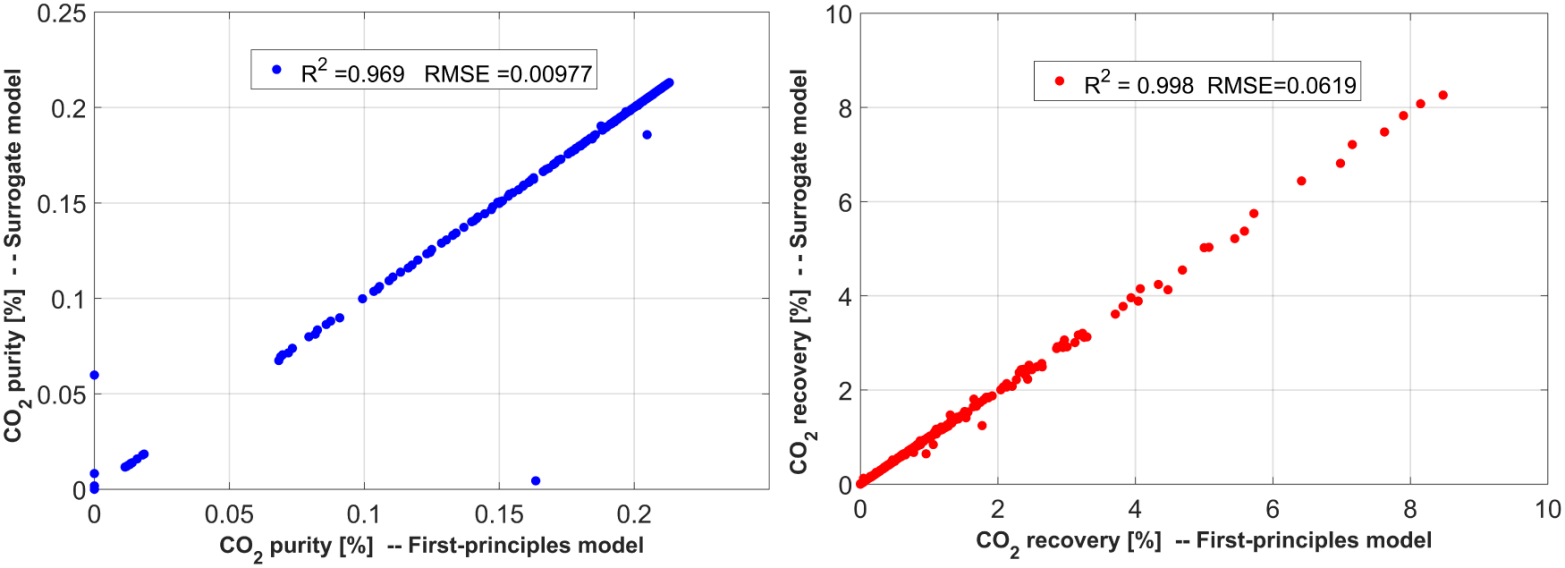
Parity plots describing the performance of the *Random Forest* regressor in capturing the 1st module CO_2_ capture behavior. Purity *R*^2^ = 0.969, RMSE = 0.0097/Recovery *R*^2^ = 0.998, RMSE = 0.0619.

The surrogate model for this case study was trained using data from the input/output mapping performed during the operability analysis of the simulated CO_2_ capture process. The ranges for the membrane properties (AIS/Inputs) are outlined in Table 3. The process operating conditions used are as shown in Tables 1 and 2. A total of 1225 data points were generated, showing the relationship between the AIS and AOS. The random forest regressor, available in *scikit*-*learn*^32^ was employed to capture the underlying relationship between inputs and outputs. The inputs and outputs were scaled and the surrogate model was generated. The parity plots for the predicted outputs and the regression performance metrics are shown in Figure 3.

##### 3.2.1.2 Case 2: Capture Operating Cost and Purity Outputs of the First Membrane Module

The second analysis focused on the cost associated with capture. The output variables for this study were CO_2_ purity and the processing cost, measured in $/ton of captured CO_2_. It is worth mentioning that the methodology used in this study to perform cost calculations is explicitly based on energy consumption and its associated cost, considering the average price of industrial electricity. While capital expenditures (CAPEX) are an essential factor in total capture cost evaluations, the objective here is to explore how material selection affects the process economics through energy-related operating costs (OPEX). Future studies will expand on this by incorporating CAPEX and OPEX considerations toward a detailed techno-economic analysis for enabling m-DAC technologies. A detailed cost breakdown has been included in the Supporting Information as well (Section S1.2). The cost of electricity used here is 0.012$/kWh according with the EIA.^33^

**Figure 4:**
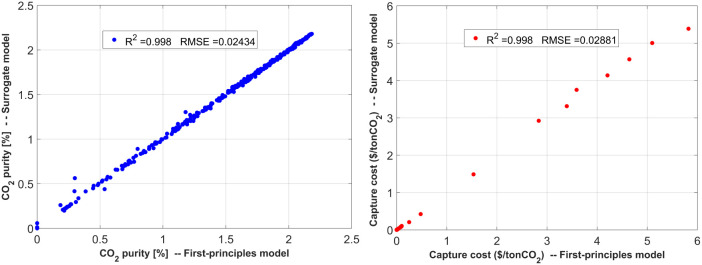
Parity plots describing the performance of the *Random Forest* regressor in capturing the 1st module CO_2_ capture related to the overall process capture operating cost behavior. Purity *R*^2^ = 0.998, RMSE = 0.024/capture operating cost *R*^2^ = 0.998, RMSE = 0.028.

Similar to the first case, 1225 data points were collected and the random forest regressor was used to generate the surrogate model. Figure 4 exhibits the parity plot and regression metrics for this case.

##### 3.2.1.3 Case 3: Recovery and Purity Outputs of the Second Membrane Module

**Figure 5:**
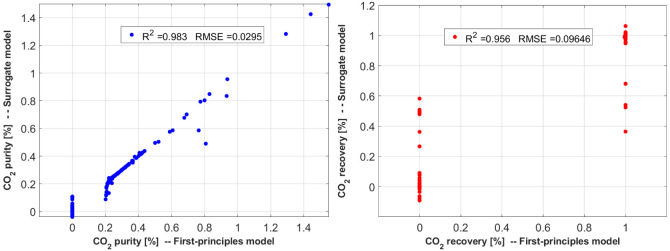
Parity plots describing the performance of the *RBF*, *Matern*, and *Rational Quadratic* regressors in capturing the 2nd module CO_2_ capture behavior. Purity *R*^2^ = 0.983, RMSE = 0.0295/Recovery *R*^2^ = 0.956, RMSE = 0.0964.

This study required a combination of three different regressors to satisfactorily reproduce the simulated conditions. Here, RBF, Matern, and Rational Quadratic regressors were combined to take advantage of their respective properties. The regression performance is shown in Figure 5.

Some outliers exist in the parity plots shown in Figures 3–5. It should be noted that the presence of these outliers in the aforementioned figures was not significant enough to compromise the quality of the predictions obtained by the surrogate models, as highlighted in Section S1.3.

### 3.3 Facilitated Transport Membrane: Parameter Regression, Simulation, and Operability Studies

In earlier work,^5^ the modeled component transport across the membrane used a general transport model, as shown in eq 3. In the present investigation, however, the FTM parameters were of interest, and to study their impact, a more accurate model was required. Therefore, leveraging the work of Xu et al. (2023)^16^ and Lee et al. (2022)^30^ the APS CO_2_ flux model was adapted to follow the facilitated transport regime assumptions, as described by eq 7.

**Figure 6:**
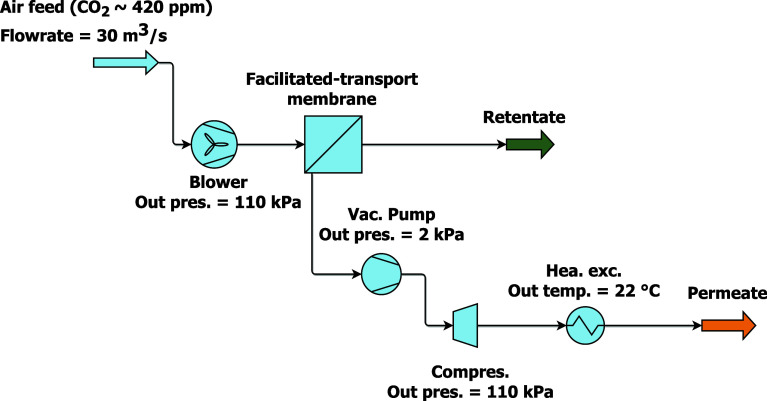
Process flow diagram of a single-step facilitated transport membrane separation for DAC, showing the feed, membrane module, and product streams.

The previous study established that the second stage can easily concentrate its feed to a range of different purities without compromising its recovery. Given this finding, focusing solely on the *first stage* significantly simplifies the modeling process. Moreover, the feed for the second stage is >1.5% in CO_2_ concentration, and under these conditions the membrane transport mechanism switches to traditional SD due to carrier saturation, as is noted in Figures S1 and S2. Figure 6 illustrates the simulated process arrangement and Table 4 highlights the new simulation parameters considering only 1 stage of separation.

**Table 4:** Facilitated Transport Model Membrane Module Parameters and Selectivity Equations

Parameter	Membrane module
Membrane module surface area [m^2^]	1000
No. of fibers	1,051,061
Fiber length [m]	1.5
Permeance [GPU]	
CO_2_	
Selectivity (30)	
CO_2_/N_2_	
CO_2_/O_2_	

**Table 5:** Tabulation of Diffusion Coefficients () and Equilibrium Constants (*K*_eq_) for CO_2_ Facilitated Transport from Prior Research

Papers	[mol/m·s]	*K*_eq_ [Pa^–1^]	Reference
This work	3.79 × 10^–11^	2.64 × 10^–5^	(30)
Yuan et al. (2011)	4.19 × 10^–9^	5.53 × 10^–6^	(13)
Zou and Ho (2006)	3.38 × 10^–12^	1.04 × 10^–1^	(34)
Xu et al. (2023)	4.50 × 10^–10^	2.00 × 10^–3^	(16)

By introducing the facilitated transport mechanism into the simulation platform, manipulating the CO_2_ carrier constant of equilibrium, *K*_eq_, and the apparent CO_2_ diffusivity, , was made possible, allowing us to conduct operability studies and inverse design on these variables. The *K*_eq_ and  parameters for the membrane assumed in this work were determined by regressing the CO_2_ permeance data from Lee et al. (2022)^30^ which included points up to 40 kPa of CO_2_ partial pressure on the feed side. The regression metrics and determined parameters are shown in Figure S1.

These parameters are the focus of the operability studies, as described in the subsequent sections.

### 3.4 Facilitated Transport m-DAC Operability Studies

The operability studies in this work, were designed to investigate the impact of material characteristics (, *K*_eq_) on the final system properties (CO_2_ purity, recovery, etc.) and the bounds of this operability analysis was based on values obtained from prior literature, *K*_eq_ and  were manipulated between the AIS bounds, as outlined in Table 6.

**Table 6:** Mapping of AIS and Desired Output Set (DOS) for FTM Operability and Inverse Design

AIS/Inputs	AOS/Outputs	DOS
*K*_eq_ [Pa^–1^]: 1 × 10^–7^–1 × 10^2^	CO_2_ purity	CO_2_ purity [mol %]: 1.0–1.75
[mol m^–1^ s^–1^]: 1 × 10^–14^– 1 × 10^–6^	CO_2_ recovery	CO_2_ recovery [%]: 50–95

In Section 5, the sparse availability of data of these parameters is discussed, aiming to push for more reporting of facilitated transport intrinsic parameters.

The AIS bounds for *K*_eq_ and  are designed to encompass a wide range of possibilities. For *K*_eq_, the range starts near the regressed values from Lee et al. (2022), as seen in Table 5, and extends to those described by Xu et al. (2023). For , due to limited reports in the literature, the AIS spans a broad range around the regressed value from Lee et al. (2022). This expanded search space evaluates numerous potential combinations of these properties, including those not yet reported or achievable, to establish a framework for material analysis in larger membrane capture processes.

Regarding the DOS bounds in this case study, the purity bounds were selected based on the results of the previous operability analysis conducted for the FTM capture. This selection followed a similar rationale to that used in the Case 1 inverse design, where the objective was to achieve the highest possible purity. The DOS range for CO_2_ recovery was proposed aiming to encompass recovery values that, when compared with results from previous studies, would exhibit higher results. Additionally, the proposed recovery bounds cover a range in which the associated purities would not significantly differ from the highest purity achievable, in order to guarantee an optimal recovery/purity pair.

#### 3.4.1 Summary of Mathematical Variables

SymbolDescriptionUnits*P*Permeabilitybarrer*l*Membrane thicknessmicron (m*10^–6^)*J*Thickness normalized fluxmol/s m^2^Δ*p*Pressure differencekPa*P*′PermeanceGas Permeation Unit
(GPU)*D*Diffusion coefficientcm^2^·s^–1^*S*Sorption
coefficientcm^3^ STP·cm^–3^·cmHg^–1^CO_2_ apparent
diffusivitymol/m·s*K*_eq_Equilibrium constantPa^–1^*f*Feed side variable-*p*Permeate side variable-*x*Mole fraction-

## 4 Results and Discussions

### 4.1 2-Staged Membrane Inverse Design Studies for Intrinsic Properties’ Mapping

**Figure 7:**
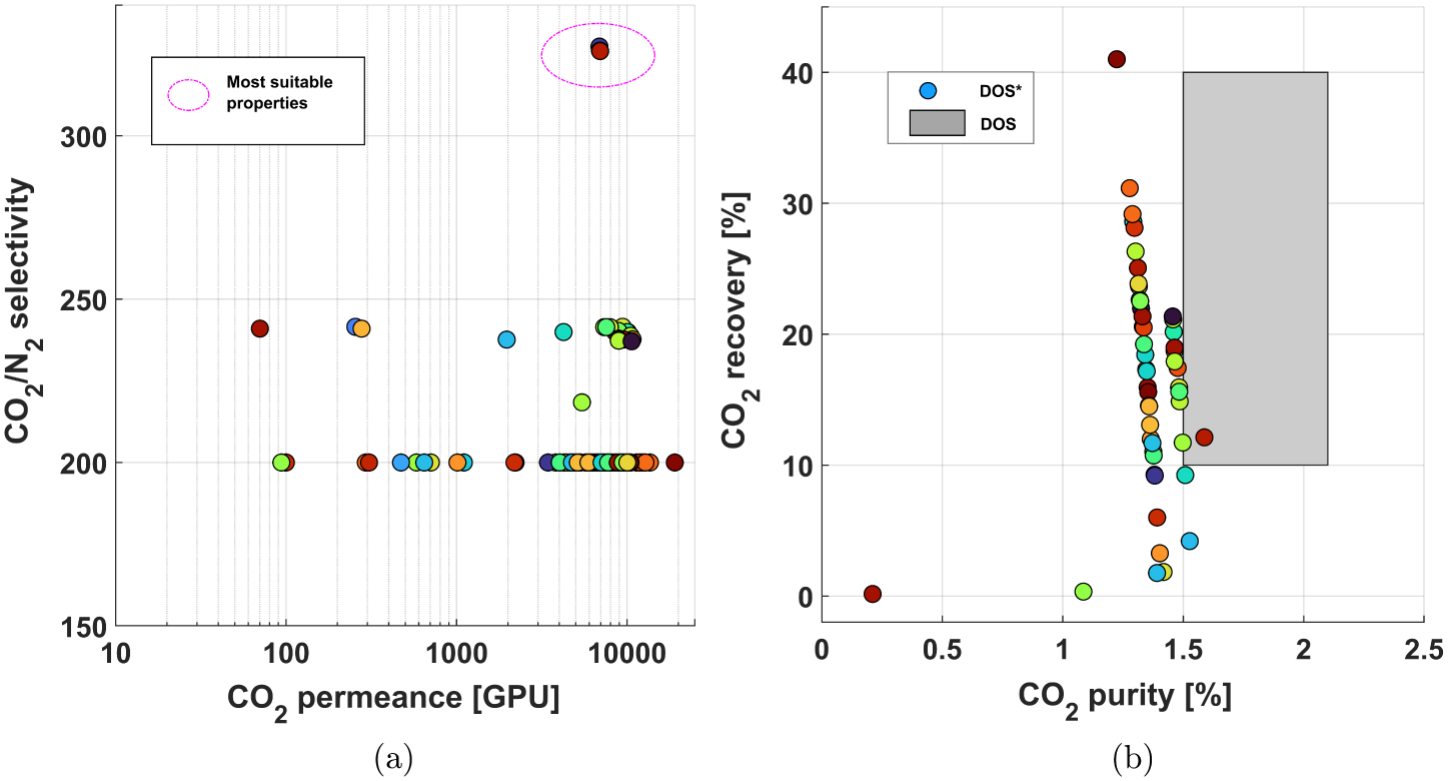
Feasible Desired Input Set (DIS) and Feasible Desired Output Set (DOS*) for Case 1. (a) DIS* representing CO_2_ permeance vs CO_2_/N_2_ selectivity, highlighting the most suitable properties. (b) DOS* showing CO_2_ purity vs CO_2_ recovery, where the shaded region represents the desired output region for the investigated parameters.

In Figure 7, the gray rectangle in Figure 7b highlights the region where the outputs meet the desired production region. In this analysis, however, only 2 points are within these boundaries. The NLP-based approach tries to minimize the distance between all achievable points and the points inside the desired region of operation. Here, the small number of points in this intersection can be explained by the separation limit imposed by the pressure-ratio limited region. The DOS could theoretically be extended to encompass a wider variety of points, allowing for a more extensive exploration of outputs. Nevertheless, this study focuses on illustrating the inverse mapping methodology, which can later be utilized to expand the scope of outputs analyzed.

The inverse design approach on the DOS helped to identify the optimum range of CO_2_ permeance and CO_2_/N_2_ selectivities, as indicated on Figure 7a. Using the proposed approach, this optimum range for the first membrane module is 6,930 GPU ± 24.8 GPU and 326 ± 0.5, for CO_2_ permeance and CO_2_/N_2_ selectivity, respectively.

**Figure 8:**
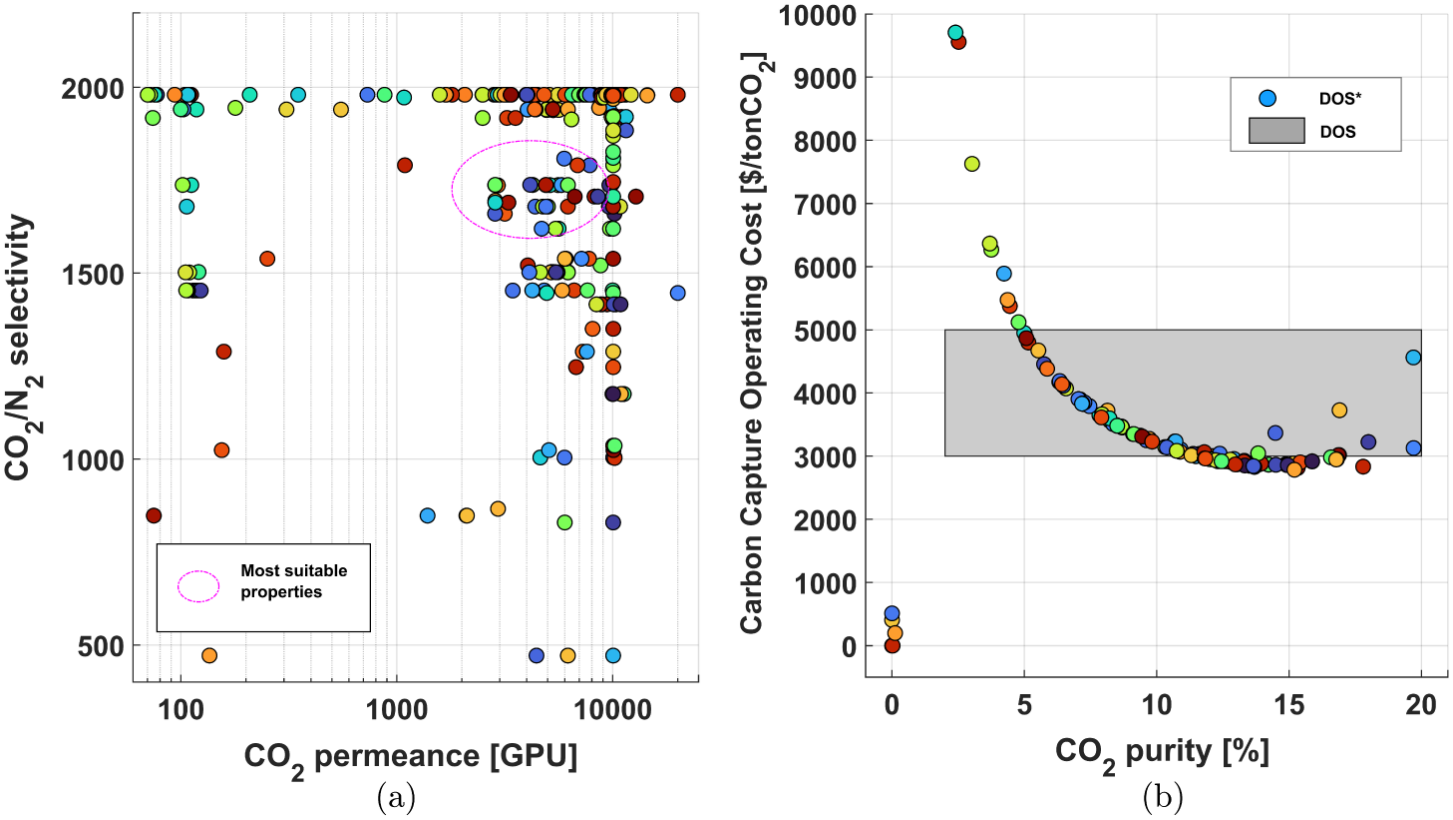
Feasible Desired Input Set (DIS) and Feasible Desired Output Set (DOS*) for Case 2. (a) DIS* shows the relationship between CO_2_ permeance and CO_2_/N_2_ selectivity, highlighting the most suitable membrane properties. (b) DOS* displays CO_2_ purity versus the carbon capture operating cost, with the shaded region indicating the desired output region for the investigated parameters.

The results presented in Figure 8 highlight the influence of intrinsic membrane properties on carbon capture costs, with a particular focus on how permeability and selectivity affect operational expenses. Although this work does not aim to provide a comprehensive economic analysis, including capital costs, it establishes a direct link between material properties and energy consumption, which is a major cost driver in m-DAC processes.

This case study exhibits several points within the desired operation region, showing a minimum trend behavior. As seen in Figure 8a, a subset of these properties (circled in pink) exhibits a combination of moderate-to-high CO_2_ permeance and high selectivity, which is desirable for minimizing compression work and energy use. This result aligns with prior literature findings as well as our earlier paper, which shows that high membrane selectivities are essential for approaching the minimum energy requirement region.^5^ For instance, in this case study, reaching the minimum cost (≈$3000/tonCO_2_) requires a CO_2_ permeance of ≈6100 GPU ± 2,150, in which the mean is fairly close to the previous study, and CO_2_/N_2_ selectivity of 1753 ± 343, which in this case is approximately 5 times greater than the selectivity from the previous case study. In the following section, the CO_2_/N_2_ selectivity and CO_2_ permeance values suggested above, for minimal energy consumption of this process, are highlighted in Figure 11a,b to correlate these intrinsic properties values with material properties of FTMs. Additionally, the findings from the inverse design studies highlight that CO_2_ purities around 5% are typically associated with CO_2_ permeances between 6,000 and 7,000 GPU. However, achieving minimal capture costs strongly depends on significantly higher selectivities. To address concerns about cost calculations, the methodology used in this study is explicitly based on energy consumption and its associated cost, considering the average price of industrial electricity. While capital expenditures (CAPEX) are an essential factor in total capture cost evaluations, the objective here is to explore how material selection affects the process economics through energy-related operational costs (OPEX). The insights gained provide a foundation for identifying membrane properties that lead to lower energy consumption and offer practical guidance for material development in m-DAC applications. Future studies could expand on this by incorporating CAPEX considerations to refine total cost assessments.

**Figure 9:**
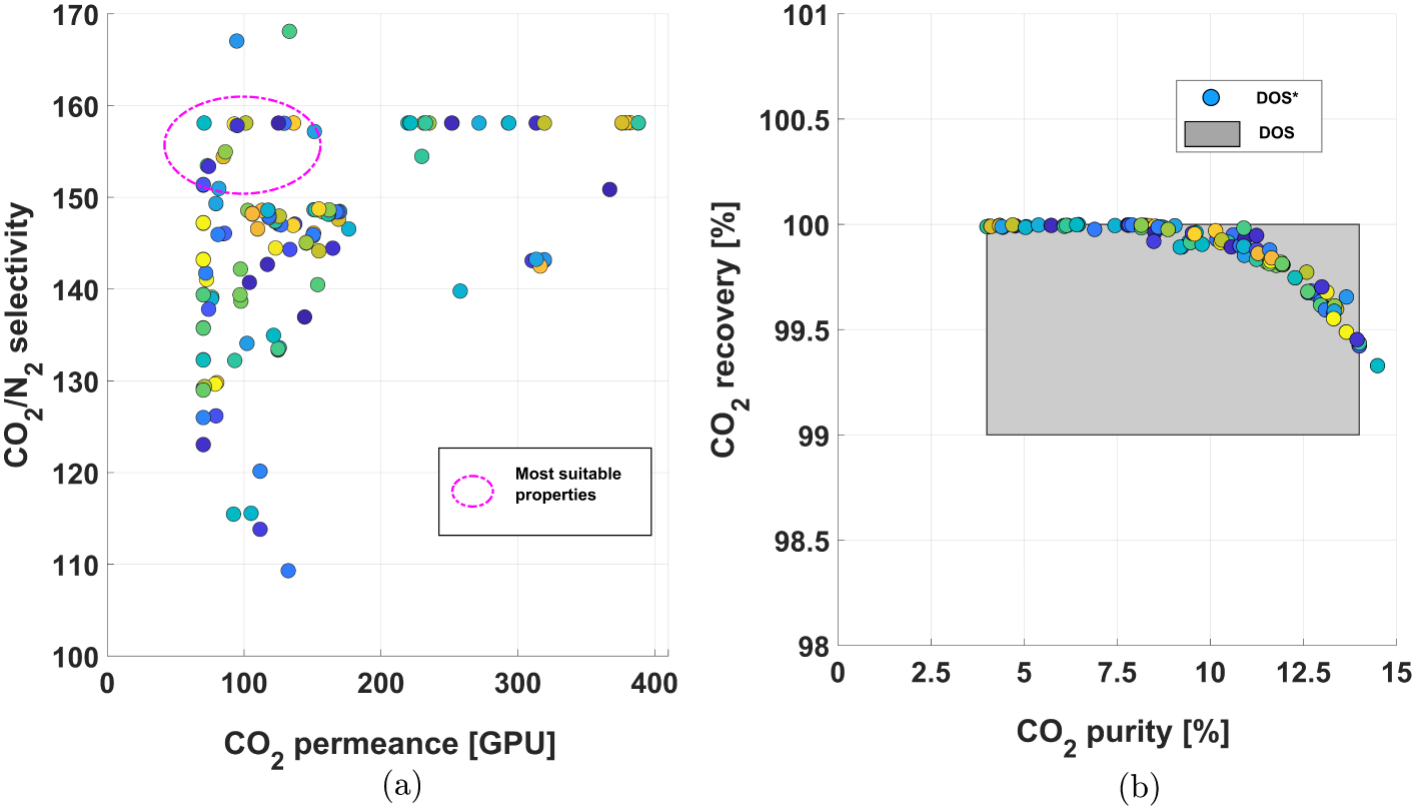
Feasible Desired Input Set (DIS) and Feasible Desired Output Set (DOS*) for Case 3. (a) DIS* represents the relationship between CO_2_ permeance and CO_2_/N_2_ selectivity, with the most suitable properties highlighted. (b) DOS* shows CO_2_ purity versus CO_2_ recovery, with the shaded region depicting the desired output region for the investigated parameters.

In case of the second module, several data points for the intrinsic properties on the feasible desired input set plot, in Figure 9, converge around CO_2_ permeance of 100 GPU. The lower CO_2_ permeance values suggest that in the second module CO_2_ separation requires less efforts to achieve higher recoveries and purities. The selectivities move upward from around 100 and collapse halfway toward the upper bound for the selectivity of this module (200). In contrast to the first case, numerous data points reach the desired region of operation for this case study. The second stage, which deals with a more concentrated feed, shows more flexibility in achieving a wider range of purities, while barely impacting its recovery.

This reinforces the fact that after the first module, the separation at the designed pressure- ratio (55) can achieve desirable purities for the capture process and maintain high recovery at the downstream separation step of the 2-staged membrane separation process at DAC conditions.

### 4.2 Facilitated Transport Assisted Capture and Inverse Design

Given the discussions of the previous section, the 2-stage separation seems to deal with more severe capture challenges in the first stage, while the second stage is shown to be much more flexible in terms of CO_2_ purity and recovery. In this section, the analysis of the facilitated transport mechanism will be applied to the first stage only, ignoring the second module since, based on previous discussions, its performance considering a simple solution- diffusion mechanism shows to be sufficient.

#### 4.2.1 Operability Studies

In the facilitated transport simulation, the flux model considers that the CO_2_ transport is facilitated by amine carriers embedded in the membrane material. The material intrinsic parameters were regressed using experimental data of the CO_2_ permeance across the membrane depending on CO_2_ feed partial pressure. The regression procedure and metrics, as well as other literature regressions, are discussed in the Supporting Information. Performing this operability study on the *K*_eq_ and  parameters can shed light on their impacts on key system properties, such as selectivity, permeance, and overall separation performance. Balancing these parameters is critical for achieving the desired trade-off between separation efficiency and energy consumption. The insights discussed in this research could aid membrane material design by guiding the development of tailored chemistries and morphologies that connect membrane intrinsic properties with process requirements.

**Figure 10:**
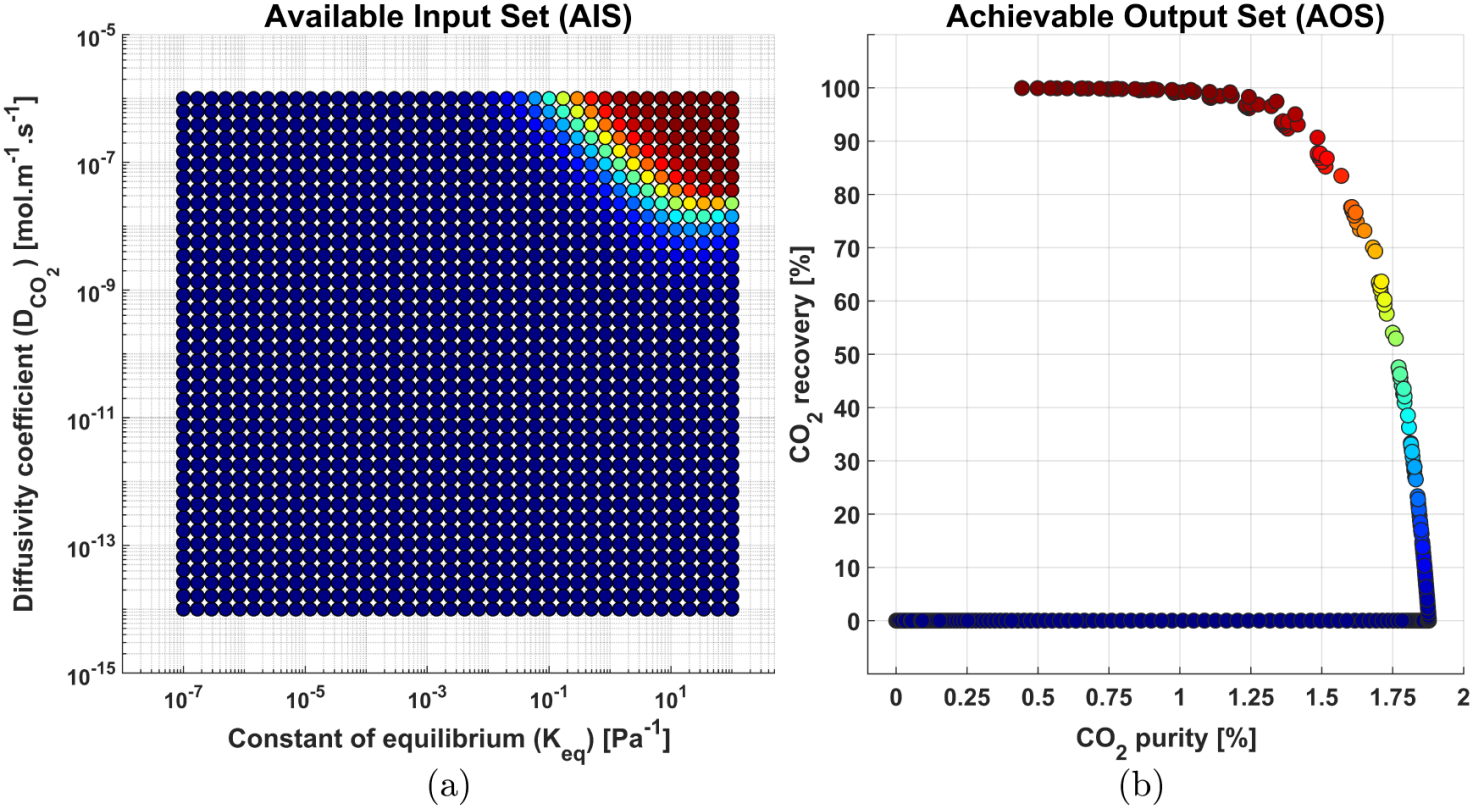
Available Input Set (AIS) and Achievable Output Set (AOS) for a facilitated-transport membrane system. (a) AIS illustrates the investigated range of equilibrium constant *K*_eq_ and CO_2_ diffusivity  values. (b) AOS presents the corresponding CO_2_ capture outputs in terms of purity and recovery.

First, an operability study was performed in order to measure the impact of the *K*_eq_ and  in the process capture, evaluated by CO_2_ recovery and CO_2_ purity. Following the color gradient exhibited in Figure 10, it is evident that most of the parameter pairs map to a low recovery region in the AOS. Nevertheless, the purity still grows up to 1.8% at its highest, 1.8% at its highest, thus achieving ≈40x feed enrichment in a single membrane pass.

The observed CO_2_ purity trends at extreme recovery values arise from the interaction between transport properties, membrane selectivity, and the imposed operational constraints. At near-100% CO_2_ recovery, the stage cut remains low (≈10%), meaning that only a portion of the total feed permeates, leading to a permeate composition that differs from the bulk feed. Conversely, at very low recovery, the reduction in CO_2_ purity is attributed to the dominance of less selective species in the permeate as the absolute amount of CO_2_ permeated becomes negligible.

The top right corner of the AIS, in Figure 10a, visually shows the color gradient changing into different colors, displaying that this region has a stronger impact on the capture. Up until  values around 10^–9^ [mol m^–1^ s^–1^], increases in constant of equilibrium only seems to improve the CO_2_ purity, demonstrating that the CO_2_ is effectively binding to the carriers, however, at a very low diffusion rate. Beyond 10^–9^ [mol m^–1^ s^–1^], for , discernible changes are observed in case of both the output variables. CO_2_ recovery begins to grow, achieving 100% in the best case scenarios, however, the purity is negatively impacted by high recovery, validating the recovery-purity trade-off relationship.

An important consideration in modeling this is the inherent trade-off that in principle should exist between equilibrium constant (*K*_eq_) and diffusivity (). In principle, a higher *K*_eq_, indicative of stronger binding between CO_2_ and carrier molecules, should result in reduced diffusivity and vice versa. This trade-off becomes relevant when considering the values that correspond to the top-right corner of the AOS. These combinations imply both high *K*_eq_ and high , which might be unrealistic based on this scenario. In this context, it should be noted that such a trade-off relationship has not been discussed in the literature.

#### 4.2.2 Impact on CO_2_ Permeance and CO_2_/N_2_ Selectivity

In Section 4.1, the CO_2_ capture metrics (CO_2_ recovery and % purity) were shown to strongly rely on the membrane separation properties (CO_2_ permeance and CO_2_/N_2_ selectivity), considering eq 5 as the flux model. In contrast, in this section the system properties are investigated as a function of the FTM material properties ( and *K*_eq_), as described by eq 7.

**Figure 11:**
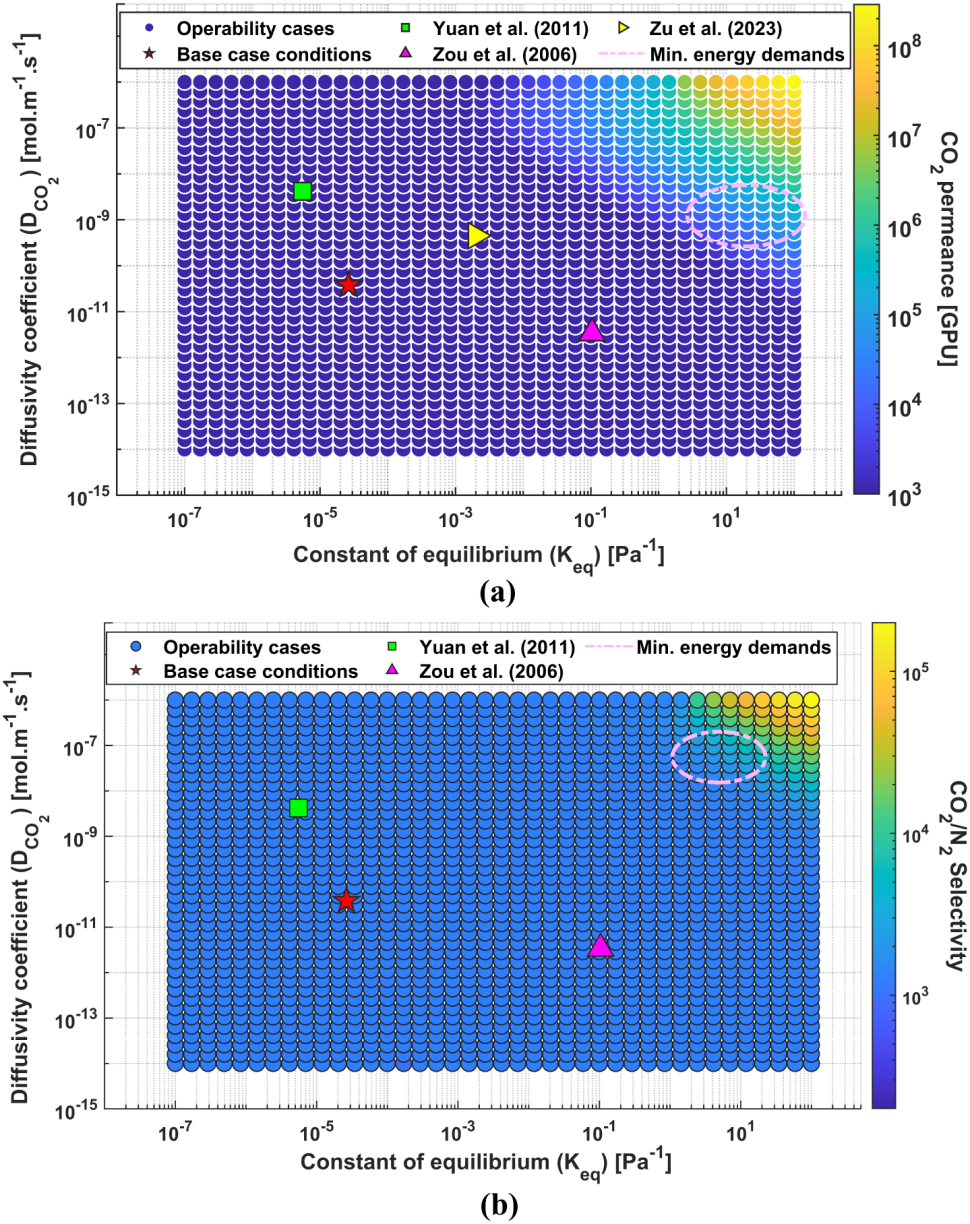
Relationship between facilitated-transport membrane parameters and separation performance. (a) The relationship between equilibrium constant (*K*_eq_) and CO_2_ diffusivity () with membrane CO_2_ permeance. (b) The relationship between *K*_eq_ and  with membrane CO_2_/N_2_ selectivity. The CO_2_ partial pressure used for this figure is ≈0.042 atm under DAC conditions.

In Figure 11, the AIS from the operability study discussed in Section 4.2.1 was correlated with the calculated CO_2_ permeance and CO_2_/N_2_ selectivity. The permeance value was determined considering the facilitated transport mechanism, as shown in eq 7, and CO_2_ pressure-gradient across the membrane material, as seen in Table 4. The N_2_ permeance, however, does not consider the FT model since the latter is only applicable for CO_2_. Ideally, the N_2_ flux would have also been modeled considering a combined flux model, as seen in eq 6, yet it was not possible to determine relevant parameters from the literature.

Here, regressed values for *K*_eq_ and  from literature were introduced to illustrate different combinations of these properties and how they affect selectivity and permeance. In general, over the designed AIS, all properties lead to similar performances as it can be observed in Figure 11a,b. The highlighted properties are located in regions where the color gradient remains consistent, indicating that the CO_2_ permeance values fall within the same order of magnitude across these areas. With respect to CO_2_ permeance, the *K*_eq_ and  fall in the range between 10^4^ and 10^5^ GPU, even though the constant of equilibrium regressed from the work of Zou et al. (2006) and the CO_2_ apparent diffusivity regressed from the work of Yuan et al. (2011) outperform the other parameters highlighted, underscoring the need for developments of membranes with enhanced properties.

Additionally, the region outlined in pink was introduced on both analyses to highlight the general area in which CO_2_ permeance and CO_2_/N_2_ selectivity values lead to minimal energy consumption ($3000/ton-CO_2_), based on the studies from Section 4.1. This stresses the importance of developing FTM materials, with enhanced properties and these results are expected to guide experimental membrane design. Specifically, CO_2_ diffusivity, which as discussed earlier, ensures high CO_2_ recoveries, could also lead to a more efficient capture. The purity of the recovered CO_2_ can be improved in the subsequent stage using a standard SD membrane.

#### *K*_eq_ and  Values Informed by Inverse Design

4.2.3

In this section, the same approach explored in Section 3.2 was applied here to accurately determine values for *K*_eq_ and  that can improve the CO_2_ recovery and purity, considering a FT membrane.

**Figure 12:**
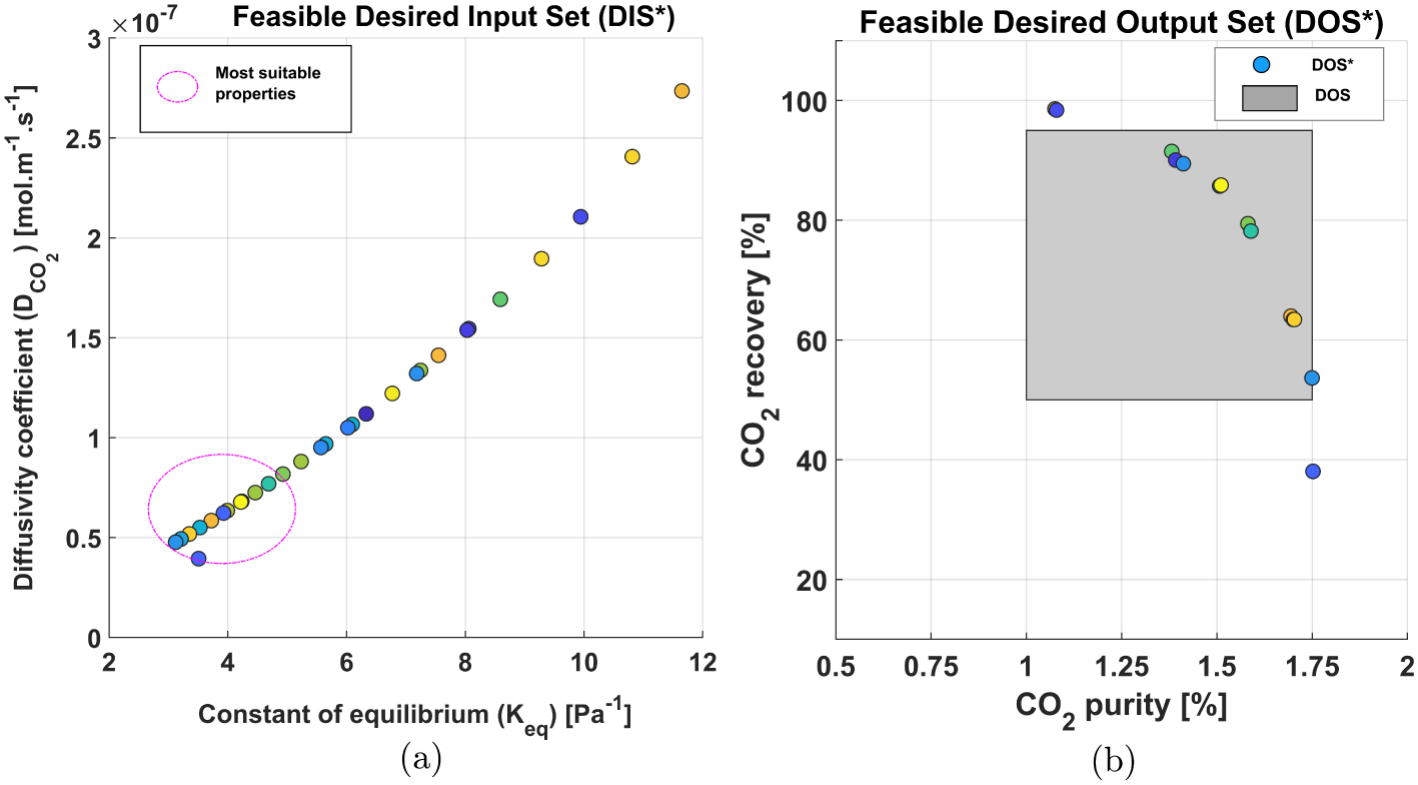
Feasible Desired Input Set (DIS) and Feasible Desired Output Set (DOS*) for the facilitated-transport membrane DAC system. (a) DIS* represents the relationship between equilibrium constant (*K*_eq_) and CO_2_ diffusivity (), highlighting the most suitable properties. (b) DOS* illustrates CO_2_ purity versus CO_2_ recovery, where the shaded region indicates the desired output region for the investigated parameters.

In the Figure 12a it is shown the material property results for the inverse design. The points that ensure operation within the targeted region result on an *K*_eq_ and  values of 4.04 ± 1.23 and 6.57 × 10^–8^ ± 2.44 × 10^–8^, respectively. However, some data points do not lead to the desired output region proposed for this study and were filtered out. Notably, there is a large difference between the properties for the base case simulation and the ones suggested by the inverse design.

This gap underscores the continued need for improved membrane materials to enable application of membrane separation for DAC. Nevertheless, both inverse designs for the 2-staged membrane separation and the FTM enabled single step separation show that using membranes, considering a large air feed (30 m^3^ s^–1^), can produce a permeate 40x more concentrate than the feed. We further provide a guidepost to membrane material developers regarding the target material and membrane properties required to improve the capture metrics.

## 5 Recommendations for Membrane Developments

To improve the accuracy and reliability of computational models for m-DAC, the following recommendations emphasize the critical role of experimental data in supporting simulation efforts and guiding future research directions:

1. **Estimation of *D*_CO_2__ and *K*_eq_ and their trade-off behavior:** The intrinsic FTM properties such as *D*_CO_2__ and *K*_eq_ clearly determine the membrane separation properties and the overall system properties, including the capture cost. However, literature data reported for these parameters is sparse. As we have demonstrated in this paper, it is possible to regress these parameters using Xu et al.’s (2023) transport model from the flux/permeance vs pressure data, but even such data are rarely reported. The model will clearly be strengthened if more experimental data are made available in the open literature.
  These two parameters are expected to have a trade-off relationship. Such trade-off relationships are very commonly reported for permeance (or permeability) and selectivity for solution-processable polymer membranes, either in an empirical form or in the form of a first-principles-based model. Our model would benefit if such trade-off relationships are reported more often for  and *K*_eq_ in the membrane literature.
2. **Estimation of *D*_N_2__, *S*_N_2__, *D*_O_2__, and *S*_O_2__:** It is a reasonable assumption that N_2_ and O_2_ do not bind with the specialized CO_2_ carriers of FTMs, and thus, *K*_eq_ for N_2_ and O_2_ can be assumed to be negligible. However, if the solution-diffusion parameters (*D*, *S*) for these cocomponents are reported, our model could also incorporate the fundamental transport behaviors of these gas molecules, and the CO_2_/N_2_ and CO_2_/O_2_ selectivities calculated would be more accurate.
3. **Estimation of properties in the presence of humidity:** The presence of H_2_O could positively or negatively impact the CO_2_ transport in FTMs. Ideally, m-DAC modeling should consider the presence of moisture in the feed. However, at this point, it is not possible to incorporate such data due to the absence of relevant experimental information in the presence of humidity. Beyond reporting CO_2_*P*/*L* and CO_2_/N_2_ and CO_2_/O_2_ selectivities in the presence of moisture, the effects on the values of  and *K*_eq_ must also be reported under these conditions, preferably over a wide range of humidity.

## 6 Conclusions

The case studies presented in this research explore different configurations and operating conditions for utilizing gas separation membranes in direct air capture (DAC) applications. The 2-stage separation analysis demonstrates that achieving useful levels of captured CO_2_ purity is highly dependent on improvements in CO_2_ permeance. However, inverse design analysis for the first separation module indicates an inherent upper bound that limits permeate purity to approximately 2%. This finding underscores the importance of refining both material properties and process configurations to overcome this limitation. In terms of cost, the minimum capture operating cost achieved remains significantly higher than the $100/tonCO_2_ benchmark set by DOE. Nonetheless, the results reveal that membranes with high selectivity can dramatically reduce capture operating costs, highlighting the importance of ongoing advancements in membrane materials to accelerate the deployment of this technology.

The inverse design analysis on CO_2_ permeance and CO_2_/N_2_ selectivity suggests that CO_2_ permeances of around 6000 GPU are sufficient to drive separation toward concentrated permeate streams, while selectivities approaching 2000 ensure feasible operation. These findings provide critical benchmarks for membrane performance.

The evaluation of facilitated CO_2_ transport membranes in this study reveals significant improvements in CO_2_ recovery during single-step separations. Manipulating key material properties, such as equilibrium constant (*K*_eq_) and CO_2_ diffusivity (), profoundly enhances recovery rates. While the operability analysis indicates that captured CO_2_ concentrations are constrained at approximately 1.8% purity under most conditions, recovery rates can reach very high levels. These results highlight the transformative potential of FTMs in improving both recovery and purity.

The FTM capture process identified optimal recovery and purity for *K*_eq_ values of approximately 4.04 ± 1.23 and  values of 6.57 × 10^–8^ ± 2.44 × 10^–8^. However, due to the limited availability of experimental data on the fabrication and scalability of these membranes, further rigorous experimental evaluations are necessary.

In addition to the multistage analysis, this study aims to bridge the gap between the intrinsic material properties of FTMs and their impact on the overall m-DAC system performance. By systematically analyzing the relationship between key parameters such as equilibrium constants (*K*_eq_) and diffusivity coefficients (*D*), and their influence on CO_2_ recovery, purity, and energy efficiency, this work establishes a framework for aligning material characteristics with process requirements. The results highlight the potential of facilitated transport mechanisms to enhance separation performance, particularly in addressing the challenges of dilute CO_2_ streams. While hurdles such as cost reduction, scalability, and achieving higher purities persist, this study provides critical benchmarks that can guide membrane material development. Future advancements in membrane materials and integrated process design can profoundly impact m-DAC technologies.

## Supporting Information Available

The experimental data and regression to determine *K*_eq_ and , the first-principles modeling background for the membrane, and CO_2_ capture costing analysis (PDF)

## References

[ref1] Negative Emissions Technologies and Reliable Sequestration: a Research Agenda; National Academies Press: Washington, D.C, 2018. 31120708

[ref2] Wilcox J.; Haghpanah R.; Rupp E. C.; He J.; Lee K. Advancing Adsorption and Membrane Separation Processes for the Gigaton Carbon Capture Challenge. Annu. Rev. Chem. Biomol. Eng. 2014, 5, 479–505. 10.1146/annurev-chembioeng-060713-040100. 24702296

[ref3] Hou R.; Fong C.; Freeman B. D.; Hill M. R.; Xie Z. Current status and advances in membrane technology for carbon capture. Sep. Purif. Technol. 2022, 300, 121863 10.1016/j.seppur.2022.121863.

[ref4] Ignatusha P.; Lin H.; Kapuscinsky N.; Scoles L.; Ma W.; Patarachao B.; Du N. Membrane Separation Technology in Direct Air Capture. Membranes 2024, 14, 30 10.3390/membranes14020030. 38392657 PMC10889985

[ref5] Gama V.; Dantas B.; Sanyal O.; Lima F. V. Process Operability Analysis of Membrane-Based Direct Air Capture for Low-Purity CO2 Production. ACS Eng. Au 2024, 4, 394–404. 10.1021/acsengineeringau.3c00069. 39185392 PMC11342364

[ref6] Baker R. W. Membrane technology and applications, 2 nd ed.; J. Wiley: Chichester, 2023.

[ref7] Rea R.; De Angelis M.; Baschetti M. Models for Facilitated Transport Membranes: A Review. Membranes 2019, 9, 26 10.3390/membranes9020026. 30717381 PMC6409752

[ref8] Chen Y.; Zhao L.; Wang B.; Dutta P.; Winston Ho W. Amine-containing polymer/zeolite Y composite membranes for CO2/N2 separation. J. Membr. Sci. 2016, 497, 21–28. 10.1016/j.memsci.2015.09.036.

[ref9] Deng L.; Hägg M.-B. Swelling behavior and gas permeation performance of PVAm/PVA blend FSC membrane. J. Membr. Sci. 2010, 363, 295–301. 10.1016/j.memsci.2010.07.043.

[ref10] Matsuyama H.; Terada A.; Nakagawara T.; Kitamura Y.; Teramoto M. Facilitated transport of CO2 through polyethylenimine/poly(vinyl alcohol) blend membrane. J. Membr. Sci. 1999, 163, 221–227. 10.1016/S0376-7388(99)00183-0.

[ref11] Yu X.; Wang Z.; Wei Z.; Yuan S.; Zhao J.; Wang J.; Wang S. Novel tertiary amino containing thin film composite membranes prepared by interfacial polymerization for CO2 capture. J. Membr. Sci. 2010, 362, 265–278. 10.1016/j.memsci.2010.06.043.

[ref12] Francisco G. J.; Chakma A.; Feng X. Membranes comprising of alkanolamines incorporated into poly(vinyl alcohol) matrix for CO2/N2 separation. J. Membr. Sci. 2007, 303, 54–63. 10.1016/j.memsci.2007.06.065.

[ref13] Yuan S.; Wang Z.; Qiao Z.; Wang M.; Wang J.; Wang S. Improvement of CO2/N2 separation characteristics of polyvinylamine by modifying with ethylenediamine. J. Membr. Sci. 2011, 378, 425–437. 10.1016/j.memsci.2011.05.023.

[ref14] Yegani R.; Hirozawa H.; Teramoto M.; Himei H.; Okada O.; Takigawa T.; Ohmura N.; Matsumiya N.; Matsuyama H. Selective separation of CO2 by using novel facilitated transport membrane at elevated temperatures and pressures. J. Membr. Sci. 2007, 291, 157–164. 10.1016/j.memsci.2007.01.011.

[ref15] Alves V.; Gazzaneo V.; Lima F. V. A machine learning-based process operability framework using Gaussian processes. Comput. Chem. Eng. 2022, 163, 107835 10.1016/j.compchemeng.2022.107835.

[ref16] Xu H.; Pate S. G.; O’Brien C. P. Mathematical modeling of CO2 facilitated transport across polyvinylamine membranes with direct *operando* observation of amine carrier saturation. Chem. Eng. J. 2023, 460, 141728 10.1016/j.cej.2023.141728.

[ref17] Fujikawa S.; Selyanchyn R.; Kunitake T. A new strategy for membrane-based direct air capture. Polym. J. 2021, 53, 111–119. 10.1038/s41428-020-00429-z.

[ref18] Wilson M. H.; Shea A.; Groppo J.; Crofcheck C.; Quiroz D.; Quinn J. C.; Crocker M. Algae-Based Beneficial Re-use of Carbon Emissions Using a Novel Photobioreactor: A Techno-Economic and Life Cycle Analysis. Bioenerg. Res. 2021, 14, 292–302. 10.1007/s12155-020-10178-9.

[ref19] Castel C.; Bounaceur R.; Favre E. Membrane Processes for Direct Carbon Dioxide Capture From Air: Possibilities and Limitations. Front. Chem. Eng. 2021, 3, 668867 10.3389/fceng.2021.668867.

[ref20] Robeson L. M. The upper bound revisited. J. Membr. Sci. 2008, 320, 390–400. 10.1016/j.memsci.2008.04.030.

[ref21] Sidhikku Kandath Valappil R.; Ghasem N.; Al-Marzouqi M. Current and future trends in polymer membrane-based gas separation technology: A comprehensive review. J. Indust. Eng. Chem. 2021, 98, 103–129. 10.1016/j.jiec.2021.03.030.

[ref22] Tong Z.; Ho W. S. W. Facilitated transport membranes for CO_2_ separation and capture. Sep. Sci. Technol. 2017, 52, 156–167. 10.1080/01496395.2016.1217885.

[ref23] Lima F. V.; Jia Z.; Ierapetritou M.; Georgakis C. Similarities and differences between the concepts of operability and flexibility: The steady-state case. AIChE J. 2010, 56, 702–716. 10.1002/aic.12021.

[ref24] Gazzaneo V.; Lima F. V. Multilayer Operability Framework for Process Design, Intensification, and Modularization of Nonlinear Energy Systems. Ind. Eng. Chem. Res. 2019, 58, 6069–6079. 10.1021/acs.iecr.8b05482.

[ref25] Georgakis C.; Uztürk D.; Subramanian S.; Vinson D. R. On the operability of continuous processes. Control Eng. Pract. 2003, 11, 859–869. 10.1016/S0967-0661(02)00217-4.

[ref26] Gazzaneo V.; Carrasco J. C.; Vinson D. R.; Lima F. V. Process Operability Algorithms: Past, Present, and Future Developments. Ind. Eng. Chem. Res. 2020, 59, 2457–2470. 10.1021/acs.iecr.9b05181.

[ref27] Alves V.; Kitchin J. R.; Lima F. V. An inverse mapping approach for process systems engineering using automatic differentiation and the implicit function theorem. AIChE J. 2023, 69, e18119 10.1002/aic.18119.

[ref28] Carrasco J. C.; Lima F. V. Novel operability-based approach for process design and intensification: Application to a membrane reactor for direct methane aromatization. AIChE J. 2017, 63, 975–983. 10.1002/aic.15439.

[ref29] Bishop B. A.; Lima F. V. Modeling, Simulation, and Operability Analysis of a Nonisothermal, Countercurrent, Polymer Membrane Reactor. Processes 2020, 8, 78 10.3390/pr8010078.

[ref30] Lee Y.-Y.; Wickramasinghe N. P.; Dikki R.; Jan D. L.; Gurkan B. Facilitated transport membrane with functionalized ionic liquid carriers for CO2/N2, CO2/O2, and CO2/air separations. Nanoscale 2022, 14, 12638–12650. 10.1039/D2NR03214G. 36040354

[ref31] Alves V.; Dinh S.; Kitchin J. R.; Gazzaneo V.; Carrasco J. C.; Lima F. V. Opyrability: A Python package for process operability analysis. J. Open Source Softw. 2024, 9, 5966 10.21105/joss.05966.

[ref32] Pedregosa F.; Varoquaux G.; Gramfort A.; Michel V.; Thirion B.; Grisel O.; Blondel M.; Prettenhofer P.; Weiss R.; Dubourg V.; et al. Scikit-learn: Machine Learning in Python. J. Mach. Learn. Res. 2011, 12, 2825–2830.

[ref33] Prices and Factors Affecting Prices - U.S. Energy Information Administration (EIA). https://www.eia.gov/energyexplained/electricity/prices-and-factors-affecting-prices.php, accessed 2025 March 19.

[ref34] Zou J.; Ho W. S. W. CO2-selective polymeric membranes containing amines in crosslinked poly(vinyl alcohol). J. Membr. Sci. 2006, 286, 310–321. 10.1016/j.memsci.2006.10.013.

