# Supplementary material for: Connecting Material Characteristics with System Properties for Membrane-Based Direct Air Capture (m-DAC) Using Process Operability and Inverse Design Approaches
Source: Ind Eng Chem Res. 2025 Apr 10;64(16):8375–89. doi: 10.1021/acs.iecr.4c04553 (PMC12022977; doi:10.1021/acs.iecr.4c04553)
Supplement: Supplementary file 1 — ie4c04553_si_001.pdf [file ie4c04553_si_001.pdf]

Connecting Material Characteristics with System Properties for Membrane-based Direct Air Capture (m-DAC) using Process Operability and Inverse Design Approaches

Vitor Gama, Deepanjali Roy, Fernando V. Lima*, and Oishi Sanyal*

Department of Chemical and Biomedical Engineering, West Virginia University, Morgantown, WV, 26506, USA

E-mail: Oishi.Sanyal@mail.wvu.edu,Fernando.Lima@mail.wvu.edu

1 Supporting Information

1.1 Experimental Data Regressions to Determine K_{eq} and D_{CO_2}

The investigation of the facilitated-transport mechanism requires the mathematical modeling of the phenomena. In this study the facilitated CO_2 flux across the membrane was modeled following Eq. equation: FT described in Xu et al. (2023) work. However, to fully model the behavior the necessary parameters, equilibrium constant (K_{eq}) and diffusivity (D_{CO_2}) had to be determined for the membrane considered as baseline for this work, which extracted from the work of Lee et al. (2022). In Xu et al. (2023), the aforementioned parameters were determined by adjusting experimental data to the flux model, however, in the work of Lee et al. (2022) the CO_2 flux as a function of the feed partial pressure was not directly reported. This relationship was key to regress the properties for the model depicted by Xu et al. (2023).[1,2] Nonetheless, the membrane baseline work reported CO_2 permeance as well as CO_2/N_2 and CO_2/O_2 selectivity trends as function of the feed partial pressure, as shown in Figures 1 and 2.

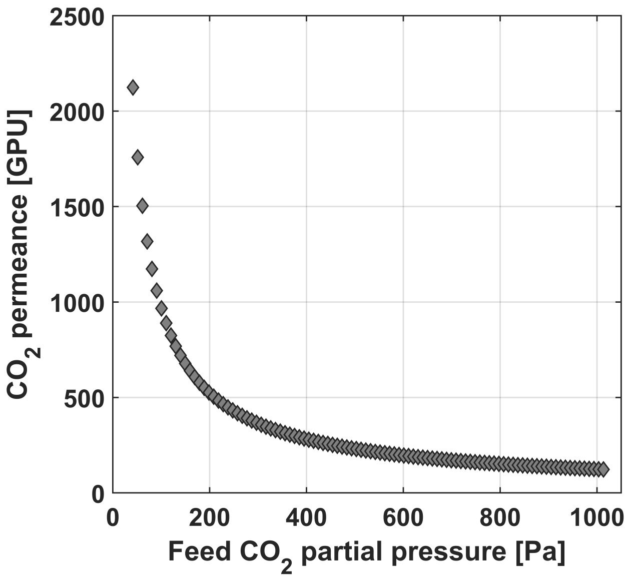

S 1: CO_2 permeance as a function of the feed CO_2 partial pressure, adapted from Lee et al. (2022)

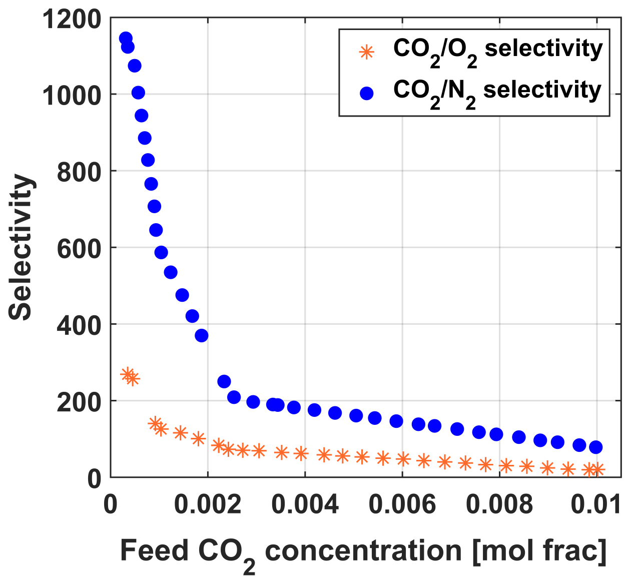

S 2: CO_2/N_2 and CO_2/O_2 selectivity as a function of the feed CO_2 partial pressure, adapted from Lee et al. (2022)

The CO_2/N_2 and CO_2/O_2 selectivity trends were regressed to a power law and used in the AVEVA Process Simulation (APS) to determine N_2 and O_2 permeances related to the CO_2 facilitated-transport flux. The CO_2 flux could be calculated by considering the relationship expressed by the following equation:

$$J_{FTMCO_2} = \frac{Q_{CO_2}}{\Delta P_{CO_2}} \quad (1)$$

Considering Eq. 1, both K_{eq} and D_{CO_2} could be regressed to the model described in Eq.

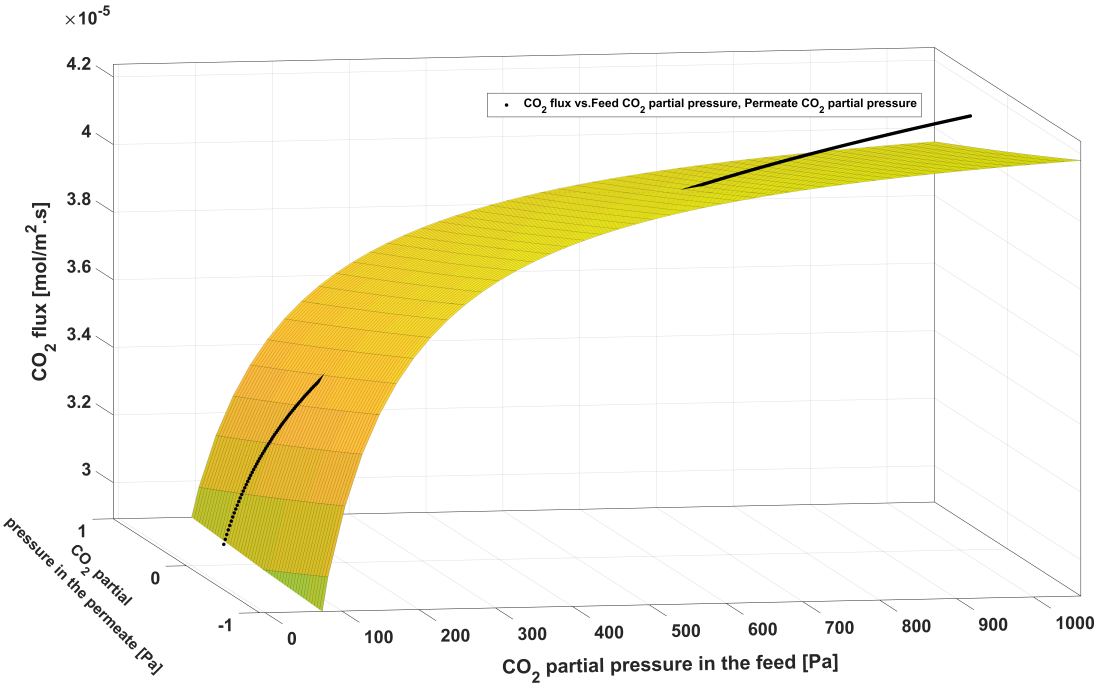

S 3: K_{eq} and D_{CO_2} regression plot, $R^2 = 0.9976$

The base case K_{eq} and D_{CO_2} determined by the regression were respectively, 2.638×10^{-5} Pa^{-1} and 3.7908×10^{-11} $mol/m^{-2}.s^{-1}$. The additional literature values were regressed following the linearization approach outlined by Xu et al. (2023).

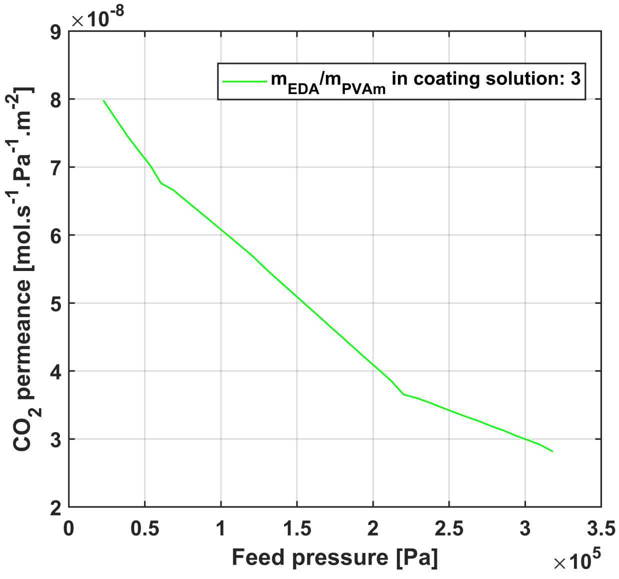

S 4: CO_2 permeance relationship with feed partial pressure for a mass ratio of EDA/PVAm in coating solution = 3, adapted from Yuan et al. (2011)

Based on the work of Yuan et al. (2011), the CO_2 flux relationship was extracted similarly to process applied for the parameter regression using the work of Lee et al. (2022).[1,3] Collecting data from Figure 4, the regression for K_{eq} and D_{CO_2} was possible. Partial pressures above 1.8×10^5 Pa were used for the regression of D_{CO_2}, while the pressures below that used to determine K_{eq}, adopting the linearization approach by Xu et al. (2023). The respective values and R^2 for each parameter is highlighted in Table 1.

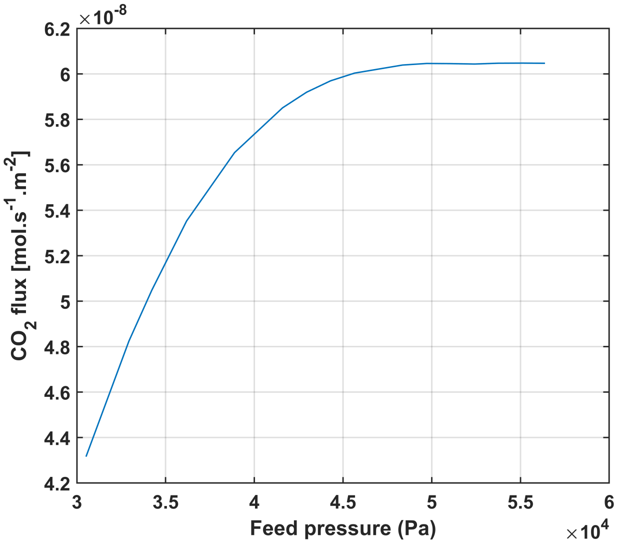

S 5: CO_2 flux relationship with feed partial pressure, adapted from the work of J. Zou and W.S.W. Ho (2006)

The data reported by J. Zou and W.S.W. Ho (2006) enabled the parameter regression

using the approach of Xu et al. (2023) straightforward, since the approach captures the behavior necessary to regress K_{eq} and D_{CO_2}.[4] In this particular case, pressures above 4.2×10^4 Pa were used to determine D_{CO_2} and the ranges until this threshold were used to calculate K_{eq}. Table 1 summarizes the regressed values for this work.

Table 1: Comparison of parameters (K_{eq} and DCO_2) and their respective R^2 values for different references.

Parameters / Reference	Yuan et al. (2011)	R^2	J. Zou and W.S.W. Ho (2006)	R^2
K_{eq} [Pa^{-1}]	5.53×10^{-6}	0.92	3.385×10^{-12}	0.67
DCO_2 [mol/s.m^2]	4.1874×10^{-9}	0.96	0.104	0.96

1.2 CO_2 Capture Operating Costing Analysis

The costing analysis for carbon capture in this membrane direct air capture (m-DAC) process was conducted using a simulation platform to evaluate the energy consumption of all pieces of the equipment involved in the proposed process. The energy demands of the equipment considered in the process include two vacuum pumps (*VP1* and *VP2*), two heat exchangers (*Hx1* and *Hx2*), two compressors (*Compress1* and *Compress2*), and one blower *B1*. These variables summed to estimate their overall energy consumption of process as follows:

$$\text{Overall energy consumption} = VP1 + VP2 + Hx1 + Hx2 + Compress1 + Compress2 + B1 \tag{2}$$

The overall energy consumption was then normalized and expressed as kilowatt-hours per ton of CO_2 ($kWh/ton\ CO_2$) captured, and later considering the average industrial electricity value (considered here to be 0.012\$/kWh),[5] the standardized \$/ton CO_2 metric for evaluating the economic feasibility of the process was calculated for each operability case ran.

1.3 Membrane Separation First-principles modeling

The membrane module considered in this model follows a shell-and-tube configuration developed by Bishop and Lima (2020).[6] In this design, the tube-side contains the feed gas, while the shell-side collects the permeate or is swept by a carrier gas. The membrane acts as a permeable barrier that allows selective transport of specific gas components based on their permeability and pressure gradients. The system operates under non-isothermal conditions, meaning heat transfer occurs between the tube and shell sides due to temperature differences, adding an energy transport component to the model.

1.3.1 Model Conventions

For the mole and energy balances, the following conventions are adopted:

1. Flow on the tube and shell sides is assumed to be in the positive axial direction.

2. The direction of permeation is assumed to be in the positive radial direction (i.e., from the tube into the shell).

1.3.2 Mole Balance Equations

1.3.2.1 Tube-Side Differential Component Mole Balance

The mole balance for a component i inside the tube accounts for:

- Flow entering at position z
- Flow leaving at position $z + \Delta z$
- Permeation loss across the membrane

At steady state:

$$\frac{dF_{i,t}}{dz} = -\pi d_t J_i \tag{3}$$

where:

- $F_{i,t}$ = Molar flow rate of component i in the tube
- d_t = Tube inner diameter
- J_i = Molar flux of component i across the membrane

1.3.2.2 Shell-Side Differential Component Mole Balance

Similarly, the mole balance on the shell side considers the gain of component i from the membrane permeation:

$$\frac{dF_{i,s}}{dz} = \pi d_t J_i \tag{4}$$

where:

- $F_{i,s}$ = Molar flow rate of component i in the shell

The direction of permeation determines the sign of J_i. If the operation is countercurrent, this equation is negative.

1.4 Membrane Transport Equation

The transport of a component across the membrane follows a pressure-driven permeation model, given by:

$$J_i = \frac{P}{l}(p_{t,i} - p_{s,i}) \tag{5}$$

where:

- P = Permeability of component i
- l = Membrane thickness
- $p_{i,t}, p_{i,s}$ = Partial pressures of component i, t in the tube and i, s in the shell, respectively

1.5 Energy Balance Equations

Energy balances account for:

1. Convective energy transport within the tube and shell
2. Energy transfer due to permeation
3. Heat exchange between the tube and shell through the membrane

1.5.1 Tube-Side Energy Balance

The energy balance for the tube side considers:

- Enthalpy flow in and out of the control volume
- Enthalpy lost due to permeation
- Heat transfer between the tube and shell

$$\frac{d(F_t H_t)}{dz} + \sum_i J_i \pi d_i \Delta z \int_{P_i}^{P_s} \left(\frac{dH}{dP}\right)_{T_i} dP + U\pi d_i \Delta z (T_f - T_s) = 0 \qquad (6)$$

where:

- F_t = Total molar flow rate of tube side
- U = Overall heat transfer coefficient
- T_t, T_s = Tube and shell temperatures
- H_t = Total molar enthalpy of tube side

1.5.2 Shell-Side Energy Balance

The shell-side energy balance accounts for energy gained via permeation and heat transfer:

$$\pm\frac{d(F_s H_s)}{dz} + \sum_i J_i \pi d_i \Delta z \int_{P_i}^{P_s} \left(\frac{dH}{dP}\right)_{T_i} dP + U\pi d_i \Delta z (T_f - T_s) = 0 \qquad (7)$$

- F_s = Total molar flow rate of shell side
- H_s = Total molar enthalpy of shell side

1.6 Surrogate models comparison with First-principles simulations

This section summarizes the case studies performed using surrogate models. Figures 6,7 and 8 show how the surrogates predicted the AOS results in comparison to the first-principle results simulated in APS.

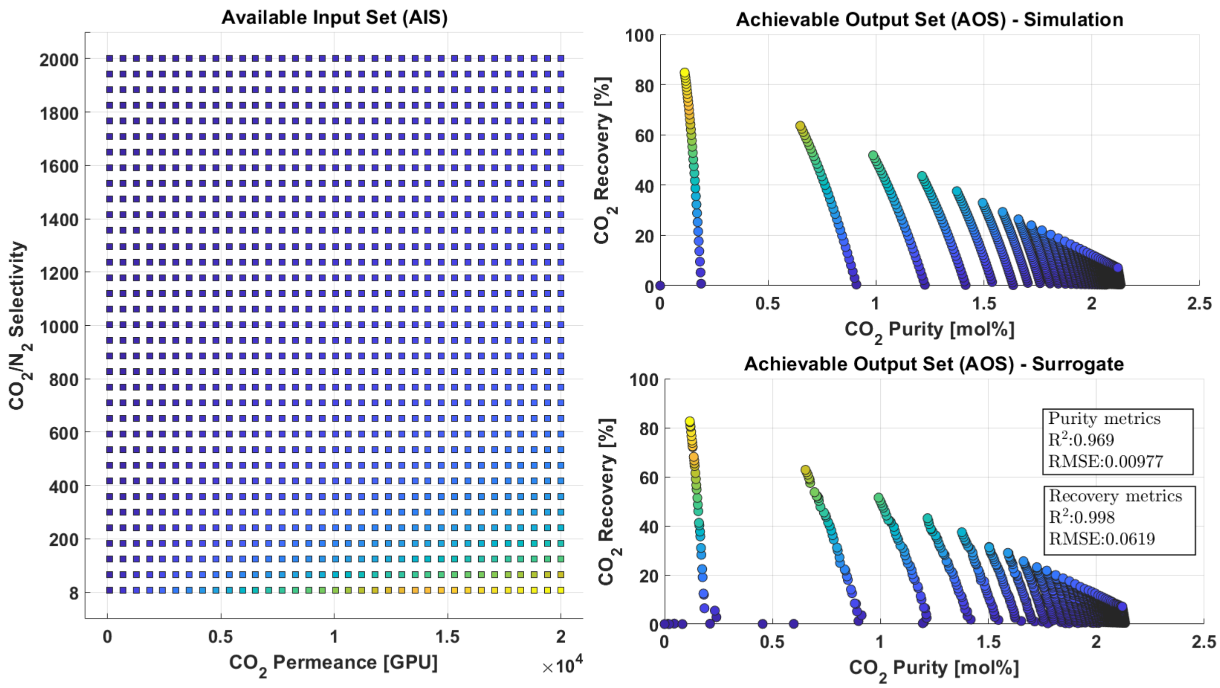

S 6: Inspection of the surrogate model trained for *Case 1*

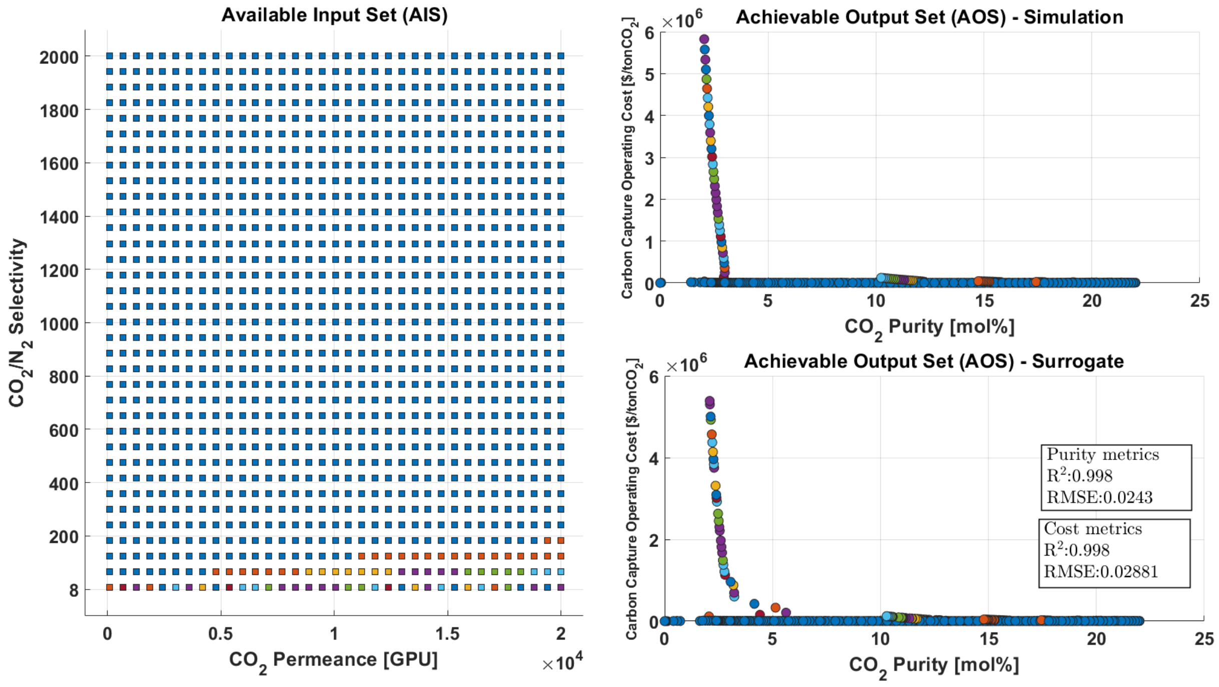

S 7: Inspection of the surrogate model trained for *Case 2*

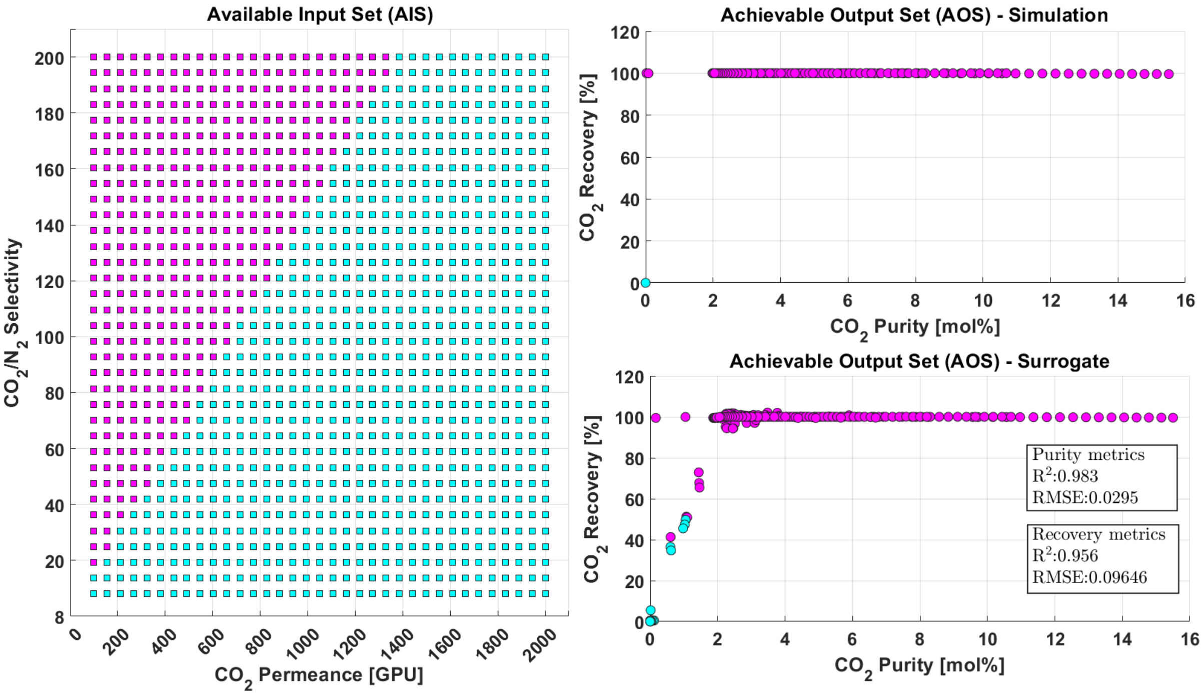

S 8: Inspection of the surrogate model trained for *Case 3*

References

(1) Lee, Y.-Y.; Wickramasinghe, N. P.; Dikki, R.; Jan, D. L.; Gurkan, B. Facilitated transport membrane with functionalized ionic liquid carriers for CO2/N2, CO2/O2, and CO2/air separations. *Nanoscale* **2022**, *14*, 12638–12650, Publisher: The Royal Society of Chemistry.

(2) Xu, H.; Pate, S. G.; O'Brien, C. P. Mathematical modeling of CO2 facilitated transport across polyvinylamine membranes with direct *operando* observation of amine carrier saturation. *Chemical Engineering Journal* **2023**, *460*, 141728.

(3) Yuan, S.; Wang, Z.; Qiao, Z.; Wang, M.; Wang, J.; Wang, S. Improvement of CO2/N2 separation characteristics of polyvinylamine by modifying with ethylenediamine. *Journal of Membrane Science* **2011**, *378*, 425–437.

(4) Zou, J.; Ho, W. S. W. CO2-selective polymeric membranes containing amines in crosslinked poly(vinyl alcohol). *Journal of Membrane Science* **2006**, *286*, 310–321.

(5) Prices and Factors Affecting Prices - U.S. Energy Information Administration (EIA). https://www.eia.gov/energyexplained/electricity/prices-and-factors-affecting-prices.php.

(6) Bishop, B. A.; Lima, F. V. Modeling, Simulation, and Operability Analysis of a Nonisothermal, Countercurrent, Polymer Membrane Reactor. *Processes* **2020**, *8*, 78.
